# A two-gene epigenetic signature for the prediction of response to neoadjuvant chemotherapy in triple-negative breast cancer patients

**DOI:** 10.1186/s13148-019-0626-0

**Published:** 2019-02-20

**Authors:** Begoña Pineda, Angel Diaz-Lagares, José Alejandro Pérez-Fidalgo, Octavio Burgués, Inés González-Barrallo, Ana B. Crujeiras, Juan Sandoval, Manel Esteller, Ana Lluch, Pilar Eroles

**Affiliations:** 1 Biomedical Research Institute (INCLIVA), Valencia, Spain; 2 Department of Physiology, Faculty of Medicine, University of Valencia, Valencia, Spain; 3 Centro de Investigacion Biomedica en Red Cancer (CIBERONC), Madrid, Spain; 4 Cancer Epigenetics and Biology Program (PEBC), Bellvitge Biomedical Research Institute (IDIBELL), Barcelona, Spain; 5 Present Address: Cancer Epigenomics, Translational Medical Oncology (Oncomet), Health Research Institute of Santiago (IDIS), University Clinical Hospital of Santiago (CHUS/SERGAS), CIBERONC, Santiago de Compostela, Spain; 6 Oncology Department, Hospital Clínico Universitario de Valencia, Valencia, Spain; 7 Pathology Department, Hospital Clínico Universitario de Valencia, Valencia, Spain; 8 Present Address: Laboratory of Epigenomics in Endocrinology and Nutrition, Instituto de Investigacion Sanitaria (IDIS), Complejo Hospitalario Universitario de Santiago (CHUS), Santiago de Compostela University (USC) and CIBER Fisiopatologia de la Obesidad y Nutricion (CIBERobn), Madrid, Spain; 9 Biomarkers and Precision Medicne Unit (UByMP), Instituto de Investigación Sanitaria La Fe (IISLaFeValencia), Valencia, Spain; 10 Physiological Sciences Department, School of Medicine and Health Sciences, University of Barcelona (UB), Barcelona, Spain; 11 Institució Catalana de Recerca i Estudis Avançats (ICREA), Barcelona, Catalonia Spain; 12 Institució Catalana de Recerca i Estudis Avançats (ICREA), Badalona, Barcelona, Catalonia Spain; 13 COST action, CA15204 Brussels, Belgium

**Keywords:** Triple-negative breast cancer, Prediction, Epigenetic signature

## Abstract

**Background:**

Pathological complete response (pCR) after neoadjuvant chemotherapy (NAC) in triple-negative breast cancer (TNBC) varies between 30 and 40% approximately. To provide further insight into the prediction of pCR, we evaluated the role of an epigenetic methylation-based signature.

**Methods:**

Epigenetic assessment of DNA extracted from biopsy archived samples previous to NAC from TNBC patients was performed. Patients included were categorized according to previous response to NAC in responder (pCR or residual cancer burden, RCB = 0) or non-responder (non-pCR or RCB > 0) patients. A methyloma study was performed in a discovery cohort by the Infinium HumanMethylation450 BeadChip (450K array) from Illumina. The epigenetic silencing of those methylated genes in the discovery cohort were validated by bisulfite pyrosequencing (PyroMark Q96 System version 2.0.6, Qiagen) and qRT-PCR in an independent cohort of TN patients and in TN cell lines.

**Results:**

Twenty-four and 30 patients were included in the discovery and validation cohorts, respectively. In the discovery cohort, nine genes were differentially methylated: six presented higher methylation in non-responder patients (*LOC641519*, *LEF1*, *HOXA5*, *EVC2*, *TLX3*, *CDKL2*) and three greater methylation in responder patients (*FERD3L*, *CHL1*, and *TRIP10*). After validation, a two-gene (*FER3L* and *TRIP10*) epigenetic score predicted RCB = 0 with an area under the ROC curve (AUC) = 0.905 (95% CI = 0.805–1.000). Patients with a positive epigenetic two-gene score showed 78.6% RCB = 0 versus only 10.7% RCB = 0 if signature were negative.

**Conclusions:**

These results suggest that pCR in TNBC could be accurately predicted with an epigenetic signature of *FERD3L* and *TRIP10* genes. Further prospective validation of these findings is warranted.

**Electronic supplementary material:**

The online version of this article (10.1186/s13148-019-0626-0) contains supplementary material, which is available to authorized users.

## Background

At the present time, chemotherapy (CT) is the only proven therapy for triple-negative breast cancer (TNBC) subtype. Anthracycline and taxane-based CT is still the standard of care for TNBC [1], with pathological complete response (pCR) rates ranging ~ 30–40% [2–6]. pCR rate in TNBC are associated with better outcomes while residual disease after neoadjuvant chemotherapy (NAC) have a higher relapse risk and poor prognosis [7]. With the advantage of high sequencing technology, several molecular signatures have been developed in the recent years to predict response to neoadjuvant chemotherapy (NAC). Oncotype-Dx [8], MammaPrint [9], Blue Print [10], Endopredict [11], or Prosigna [12] are some of them. Recently, an initial 199-gene signature, (*E2F4* target gene signature), has shown accurate prediction of response to NAC even when reduced to 33-gene panel and has been validated in 1129 patients across five independent data sets [13]. However, all these predictive panels have shown to do better in ER positive breast cancer than in ER negative. Thus, accurate prediction of response in TNBC still remains a medical need. Other study addressed the issue of the prediction to NAC in 94 patients TNBC treated with paclitaxel and carboplatin according to the Lehman’s TNBC type 4 classification. Basal-like 1 (BL1) subtype showed a pCR rate of 65.2%, while basal-like 2 (BL2) was 47.4%, mesenchymal (M) 36.4%, and luminal androgen receptor (LAR) 21.4% [14]. The I-SPY 2 is an ongoing prospective trial of NAC in breast cancer including a cohort of TNBC patients. A 70-gene panel combined with DNA deficient biomarkers have shown a 75% pCR in a subset of TNBC patients treated with carboplatin and veliparib in a recent publication of this cohort [15]. All these panels have been designed based on arrays of gene-expression techniques. Nevertheless, epigenetic modifications of certain genes can lead to silence or activation of different genes [16, 17].

The epigenetic modifications of the DNA, such as methylation, can modulate gene expression with no DNA sequence modification and contribute to disease development [18]. In this context, epigenetic changes in tumor DNA before CT administration could potentially have a predictive role of response to this therapy [18]. The aim of this study was to identify a predictive epigenetic signature of pCR as defined by the residual cancer burden (RCB) index by Symmans et al. [19](RCB = 0) in patients with TNBC treated with NAC including anthracyclines and/or taxanes-based regimens.

## Results

### Clinical characteristics of TNBC patients

Fifty-four patients were included: 24 in the discovery cohort (DC) and 30 in the validation cohort (VC). The clinical characteristics of the patients are summarized in Table 1. After biopsy, all patients were treated with NAC based on a taxane and/or anthracycline regimen and all of them were considered TNBC according to immunohistochemistry for ER, PR, and HER2. Patients were classified in responders (R) if RCB = 0 or non-responders (NR) if RCB > 0.

**Table 1:** Clinical characteristics of TNBC patients included in the study

Variable	Whole cohort *N* = 54	Discovery cohort *N* = 24	Validation cohort *N* = 30
Age; median (range)	47.88 (27.19–78.92)	46.88 (30.33–78.07)	48.49 (27.19–78.92)
cT			
cTx	5 (9.3%)	2 (8.3%)	3 (10.0%)
cT1–2	40 (74.1%)	16 (66.6%)	24 (80.0%)
cT3–4	9 (16.6%)	6 (25.1%)	3 (10.0%)
cN			
cNx	4 (7.4%)	2 (8.3%)	2 (6.7%)
cN0	32 (59.3%)	14 (58.4%)	18 (60.0%)
cN+	18 (33.3%)	8 (33.3%)	10 (33.3%)
Ki67 in biopsy			
Missing value	10 (18.5%)	7 (29.2%)	3 (10.0%)
ki67 < =50%	17 (31.6%)	7 (29.2%)	10 (33.3%)
ki67 > 50%	26 (49.9%)	10 (41.6%)	17 (56.7%)
Type of NAC			
Taxanes	14 (25.9%)	5 (20.8%)	9 (30.0%)
Anthracyclines	1 (1.9%)	0 (0%)	1 (3.3%)
Taxanes and anthracyclines	39 (72.2%)	19 (79.2%)	20 (66.7%)
RCB			
RCB = 0	19 (35.2%)	10 (41.7%)	9 (30.0%)
RCB > 0	35 (64.8%)	14 (58.3%)	21 (70.0%)
RCB:			
RCB = 0	19 (35.2%)	10 (41.7%)	9 (30.0%)
RCB = 1	8 (14.8%)	3 (12.5%)	5 (16.7%)
RCB = 2	19 (35.3%)	6 (25.0%)	13 (43.3%)
RCB = 3	8 (14.8%)	5 (20.8%)	3 (10.0%)

(*NAC* neoadjuvant treatment)

### Analysis of DNA methylome in responder and non-responder patients: discovery cohort

**Fig. 1:**
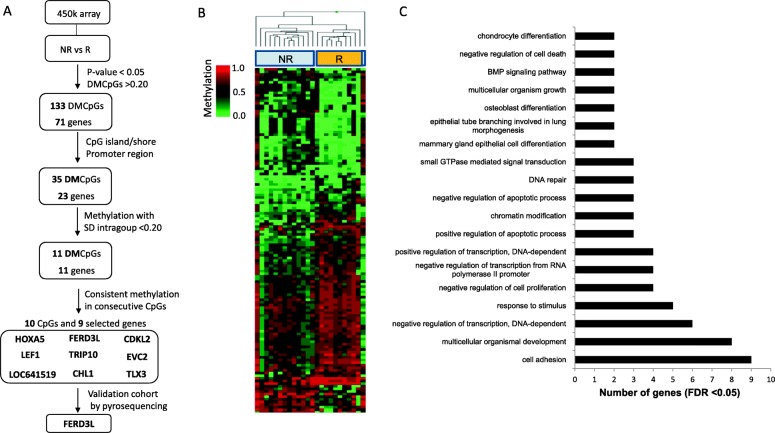
Differentially methylated CpG sites from 450K array (Illumina) between the responder and non-responder group. **a** Workflow summary. **b** Hierarchical clustering heatmap of differentially methylated CpGs: 133 CpGs (71 genes) differentially methylated were found between responders (R) and non-responders (NR) group identified from 450K array analysis with mean differences in methylation levels ≥ 20% (*p* < 0.05). **c** Most representative biological processes of the 71 differentially methylated genes according to Gene Ontology analysis

A genome-wide DNA methylation study was performed in the DC (*N* = 24, 10 R (RCB = 0) and 14 NR (RCB > 0)). Figure 1a summarizes the whole process during this study. The analysis of methylation data showed 133 CpGs sites (71 genes) with differences in methylation levels ≥ 20% (*p* value < 0.05) that distinguished R patients (treatment sensitive) from NR patients (treatment resistant) (Fig. 1b). According to a Gene Ontology (GO) analysis, some of these genes were involved in biological functions and pathways such as DNA repair, cell adhesion, transcription regulation, or signaling mediated by GTPases (Fig. 1c, Additional file 1: Table S1) that have shown to be implicated in chemoresistance of cancer, including the response to anthracyclines and/or taxanes [20–23].

**Fig. 2:**
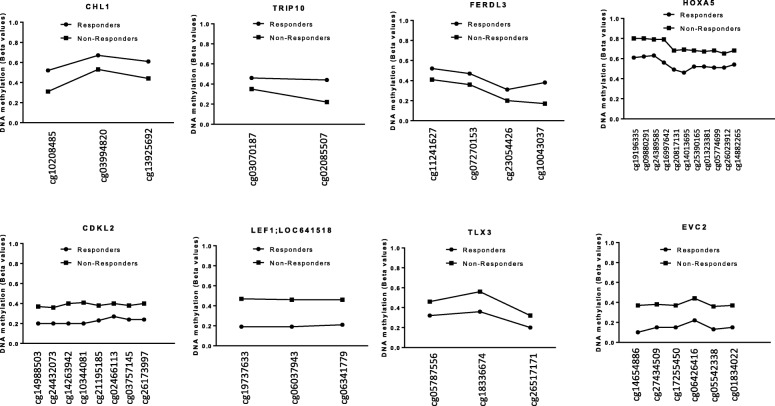
Methylation profile of the selected CpGs in responder and non-responder group in the discovery cohort (DC). From the 450K array, ten CpGs corresponding to nine genes were found in CpG islands or in the promoter regions with a standard deviation intragroup ≤ 20% and with a consistent DNA methylation profile of consecutive CpGs. These candidate genes were *LOC641519*, *LEF1*, *HOXA5*, *EVC2*, *TLX3*, and *CDKL2* with high methylation in non-responder group and genes *FERD3L*, *CHL1*, and *TRIP10* with high methylation in responder group. Consecutive CpGs to the CpGs of interest were evaluated in each gene by the 450K array. Points represent mean methylation values from patients

Thirty-five CpGs located in promoters, islands, or shores from 23 genes were selected to further validations (Additional file 2: Table S2). Of these, taking into account an intra-group SD  ≤0.2, 11 CpGs corresponding to 11 genes showed significant methylation differences (Additional file 3: Table S3), and 9 of these genes showed a consistent methylation profile on consecutive CpGs (Fig. 2). These candidate genes were LOC641519, *LEF1*, *HOXA5*, *EVC2*, *TLX3*, and *CDKL2* with high methylation in NR group and genes *FERD3L*, *CHL1*, and *TRIP10* with high methylation in R group.

### Validation of methylation in candidate genes by pyrosequencing: validation cohort

A pyrosequencing study in the DC and in the VC (*N* = 30, 9 RCB = 0 and 21 RCB > 0) was performed to validate the candidate genes first technically and secondly in an independent cohort, respectively. Methylation was analyzed for each gene taking into account the differentially methylated CpGs identified in the 450K array and other CpGs located close to them in order to obtain a more consistent result (Additional file 4: Table S4).

**Fig. 3:**
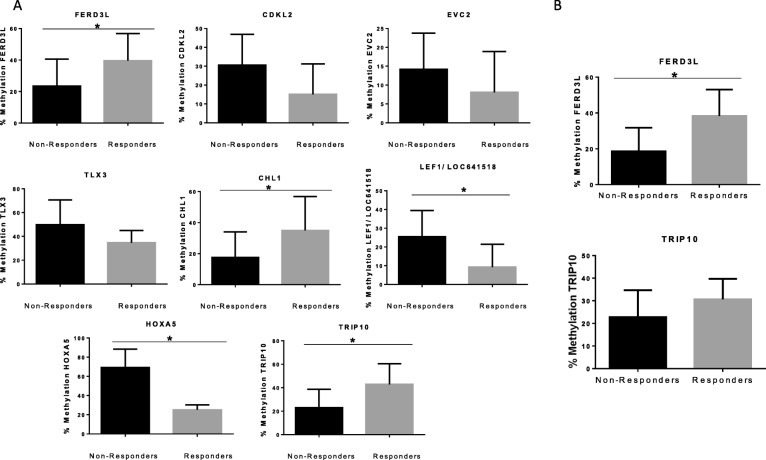
Validation of the differently methylated CpGs by pyrosequencing. **a** Validation results in the discovery cohort (DC, *n* = 24)). The array data were replicated in *LEF1/ LOC641519* and *HOXA5* genes*,* with a significant higher methylation in non-responders patients vs. responder patients (*p* < 0.05) and *FERD3L*, *TRIP*, and *CHL1* genes, with a significantly higher methylation in responder patients vs. non-responder patients (*p* < 0.05). The *CDKL2*, *EVC2*, and *TLX3* genes showed a trend for significance (*p* ≥ 0.05). **b** Validation results in the validation cohort (VC, *n* = 30). Methylation data from the 450K array were only replicated for *FERD3L* gene. Values were statistically different when compared non-responder vs. responder group showing low methylation in non-responder patients (*p* = 0.0087). The *TRIP10* gene showed a non-significant trend towards a high methylation in responder group compared to non-responder group (*p* = 0.19)

In the DC, we replicated by pyrosequencing the methylation data obtained in 450K array for *LOC641519/LEF1* gene (*p* value = 0.02) and *HOXA5* gene (*p* value = 0.0001), where we also observed a methylation level significantly higher in NR patients than R patients, and in *FERD3L* gene (*p* value = 0.04), *TRIP10* (*p* value = 0.003), and *CHL1* (*p* value = 0.03), where methylation was also significantly higher in R patients than NR patients. However, in *EVC2* gene (*p* value = 0.07), *CDKL2* gene (*p* value = 0.05), and *TLX3* gene (*p* value = 0.07), replication was not statistically significant but showed a trend towards a higher level of methylation in NR patients (Fig. 3a).

Pyrosequencing in the VC validated the results for *FERD3L* gene (*p* value = 0.0087) with high methylation in R group versus NR group (Fig. 3b). Moreover, differences in *TRIP10* methylation showed a trend towards significance between R and NR (*p* value = 0.19). Accordingly, by means of a biological pathway analysis of these genes using the publicly available resource *Pathway Commons* [24], we observed that *FERD3L* and *TRIP10* are able to interact with several genes (Additional file 5: Figure S1) which have been previously associated with therapy resistance of breast cancer and other types of tumors [23, 25].

### *FERD3L* methylation and gene expression in TNBC cell lines

**Fig. 4:**
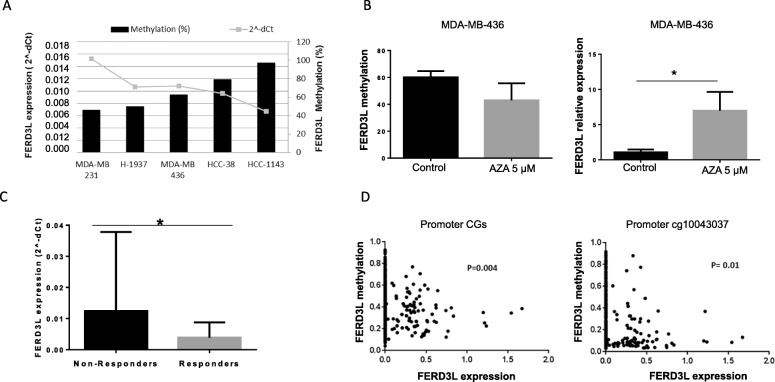
*FERD3L* methylation and transcript levels in patients and cell lines. **a** *FERD3L* gene expression levels (qRT-PCR) and methylation levels (pyrosequencing) in a set of TNBC cell lines. *FERD3L* was methylated in all the cell lines studied (≥ 40%) correlating with the gene expression detected. **b** Variation in *FERD3L* methylation and gene expression in MDA-MB-436 cell line after AZA (5 uM) treatment. Cells showed a decrease in methylation level (*p* = 0.05) that correlated with a statistically significant increase in gene expression (*p* = 0.0022) when they were treated with AZA and compared with control cells not treated with AZA agent. **c** qRT-PCR results for *FERD3L* gene in TNBC patients (DC + VC). Non-responder group showed higher gene expression levels than responder group (*p* = 0.04) correlating with methylation levels obtained both in 450K array and pyrosequencing. **d** Spearman correlation between methylation and gene expression for *FERD3L* gene in a population of breast cancer patients (*n* = 713) from TCGA database. The result showed a correlation between a high gene methylation and a low gene expression when analyzed all the CpGs in the *FERD3L* gene promoter or only the cg10043037 identified in the 450K array

In order to evaluate whether DNA methylation has a functional role in the transcriptional control of *FERD3L*, we evaluated *FERD3L* gene expression by qRT-PCR and methylation by pyrosequencing in a set of TNBC cell lines (Fig. 4a) with the aim of correlating *FERD3L* methylation levels with gene expression level and corroborate the results from patients. We observed that *FERD3L* gene was methylated in all the cell lines studied with levels always higher than 40%. The *FERD3L* expression inversely correlated with the methylation detected as was expected, showing a low gene expression when methylation was high. Thereby, MDA-MB-231 cell line showed the lowest methylation level and correlated with the highest level of gene expression. Conversely, HCC-1143 cell line that showed the higher methylation was the one with the lowest gene expression level.

### *FERD3L* demethylation assays and gene expression in TNBC cell lines

In order to check if changes in *FERD3L* methylation status also affect to gene expression level, we performed an assay with the MDA-MB-436 cell line treated with AZA demethylating agent. We observed that the treatment modified *FERD3L* methylation in MDA-MB-436 cell line inducing a decrease when compared with control cells (MDA-MB-436 cell line not treated with AZA) (*p* value = 0.05). As we expected, this change in *FERD3L* methylation was correlated with a significant increase (*p* value = 0.0022) in *FERD3L* expression (Fig. 4b).

### High *FERD3L* expression levels correlates with low gene methylation in TNBC patients

The analysis of *FERD3L* gene expression in the 54 patients showed a significant difference (*p* value = 0.04) in *FERD3L* gene expression with high expression in NR patients versus R patients (Fig. 4c). Therefore, it suggests an inverse correlation between methylation and gene expression in NR patients and R patients. The Cancer Genome Atlas dataset (TCGA) analysis for 713 breast cancer patients showed negative correlation between methylation and expression, according with our data. It was detected both when all CpGs in the *FERD3L* gene promoter were included and when only analyzed the CpG cg10043037 validated for *FERD3L* gene in the study (Fig. 4d).

### Statistical model to predict response to neoadjuvant treatment in TNBC patients

Due to *FERD3L* and *TRIP10* showed the higher level of significance in the VC, we selected both genes for therapy response analysis. It is interesting to note that these two genes did not show statistical differences in methylation between R and NR in terms of age, tumor size, nodule affectation, or ki67 expression (Additional file 6: Table S5) indicating that these genes were not associated with any relevant clinicopathological prognostic factor. Importantly, we were able to create a statistical epigenomic predictive model of pathological response (RCB = 0) with the *FERD3L* and *TRIP10* methylation and using the whole cohort. These two genes were selected as both showed the higher level of significance in the VC. The statistical model for the prediction of therapy response was based on the Akaike information criterion (AIC) and by constructing a receiver operating characteristic (ROC) curve. Based on this model, the following rule was constructed:

*A* × FERD3L methylation level (%) + *B* × TRIP10 methylation level (%) > 971 (*A* and *B* being constants)

**Fig. 5:**
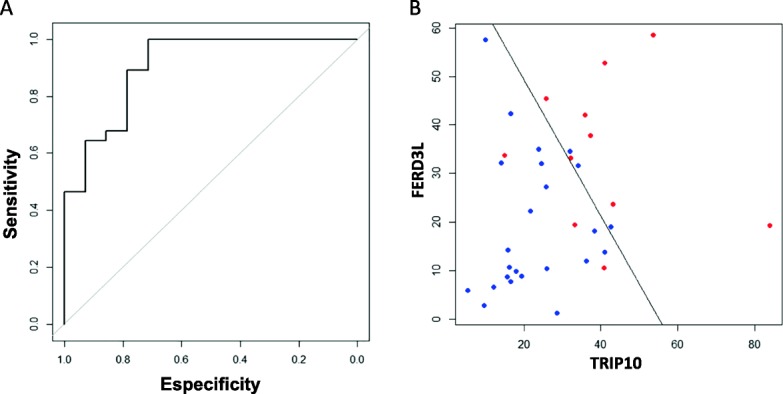
*FERD3L* and *TRIP10* genes as predictive markers of pathological complete response (pCR) in TNBC patients. **a** The ROC curve for *FERD3L* and *TRIP10* show graphically the connection/trade-off between clinical sensitivity and specificity. The area under the ROC curve (AUC) was 0.905 (95% CI = 0.805–1.000). **b** Based on the algorithm of the methylation status of *FERD3L* and *TRIP10* in patients with TNBC, when the score was > 971, the probability to get a RCB = 0 was of 78.6%. For values lower than 971, the probability to get a RCB = 0 was of 10.7%. Red points represent patients with clinical RCB = 0 and in blue are indicated those patients with clinical RCB > 0. To the right of the line, patients has been classified as RCB = 0 and to the left as RCB > 0 by our algorithm

Levels > 971 showed a high likelihood for RCB = 0 (8 out of 11 cases 78.6%), while levels  ≤ 971 showed a poor probability for RCB = 0 (3 out of 28 patients 10.7%). Value based of the calculation of the ROC curve with AUC = 0.9056 (95% CI = 0.805–1.000) (Fig. 5a, b).

## Discussion

This study evaluated the methylation profile of triple-negative breast cancer (TNBC) patients treated with neoadjuvant systemic chemotherapy (NAC) depending on the treatment response and identified a two-gene epigenetic signature for discriminating responders (R) from non-responders (NR). The discovery cohort included 24 TNBC patients and was analyzed by using Infinium Human Methylation 450 BeadChip array whereas the validation cohort included 30 TNBC patients and was analyzed by pyrosequencing for the significant and most relevant genes identified in the discovery cohort. For each case, DNA was obtained from core biopsies before the beginning of the treatment. The analysis of the discovery cohort identified nine genes differentially methylated. Six genes with higher methylation in NR patients (*LOC641519*, *LEF1*, *HOXA5*, *EVC2*, *TLX3*, *CDKL2*) and three genes with greater methylation in R patients (*FERD3L*, *CHL1* and *TRIP10*). After technical and analytical validation by pyrosequencing in both discovery and validation cohorts, we identified a two-gene (*FERD3L* and *TRIP10*) signature able to predict response to NAC. The role of promoter methylation in the regulation of *FERDL3* gene expression was evaluated in TNBC cell lines and in TNBC tissues, demonstrating an inverse correlation between methylation and expression levels. To our knowledge, the two genes epigenetic model shown in this study is the first epigenetic signature for prediction of response to NAC in TNBC patients. It should be noted that this model almost doubles the predictive potential described for the TNBC subtype by other approaches (~ 30–40% versus 78.6% with our model) [2–6].

Pathological complete response (pCR) is an effective surrogate marker for survival among patients with luminal B/HER2 negative, HER2 positive, and TNBC tumors. The results of a meta-analysis of 6377 patients with operable or advanced non-metastatic disease from six prospective neoadjuvant studies support this [26]. In the TNBC group of 911 patients who received anthracycline and taxane based NAC, 31% achieve pCR. Most recently, a retrospective analysis of 452 TNBC patients showed a pCR of 33% [3]. Overall and disease-free survival were significantly longer among patients achieving pCR versus residual disease [3]. In this context, the development of a more accurate predictive signature in TNBC could have an important clinical impact. To date, available gene panels for prediction of pCR to NAC are based in genomic signatures [8–10, 13, 27].

Epigenetics changes affect cellular processes such as gene expression and have clearly been related to the development of diseases such as breast cancer. Few methylation studies have been specifically done on TNBC subtype. Stizarken et al. identified differentially methylated regions that could separate TNBC and non-TNBC patients and classified those according to prognosis. This provided the first evidence that changes in the methylation profile of DNA could be useful to identify and stratify TNBC patients [28]. Recently, Mathe et al. performed a study of gene expression and DNA methylation in the same population, demonstrating that DNA methylation contributes to the deregulation of gene expression [29]. In this context, our results suggest that response to NAC can be predicted accurately with an algorithm of the methylation status of *FERD3L* and *TRIP10* genes in patients with TNBC.

The *FERD3L* (Fer3-like bHLH transcription factor) gene, also named *NATO3* or *N-TWIST*, is a gene located on chromosome 7 and is a basic helix-loop-helix (bHLH) transcription factor. These factors play an essential role in multiple developmental processes, mainly in neurogenesis, where its regulation is essential for the right development [30]. *FERD3L* is a member of the TWIST genes family that is implicated in epithelial-mesenchymal transition (EMT) in cancer cells, a process also related to metastasis and may lead to chemo-resistance in TNBC [31].

The TWIST genes induce cell dedifferentiation and cell migration [32] and are also related to the inhibition of apoptosis [33], the cancer stem cell phenotype [34], and chemotherapy resistance [35].

Several studies have shown that in metastatic carcinomas including aggressive and metastatic breast cancer, there are an overexpression of *TWIST* [36]. The inactivation of *TWIST* by siRNA technology or chemotherapeutic approaches has proved successful [36–38], so it is presented as a potential therapeutic target for metastatic breast cancer. There is only one work in the literature that relates the *FERD3L* gene to cancer, specifically neuroblastoma [39]. Promoter CpG islands in *FERD3L* gene was found to be highly methylated in neuroblastoma cell lines causing gene silencing and poor prognosis. However, to our knowledge, there is no previous evidence of relation between *FERD3L* expression and response to CT in the clinical setting or any relation with breast cancer.

The *TRIP10* (thyroid hormone receptor interactor 10 gene) is located in chromosome 19 and belongs to the minor histocompatibility antigens family and codifies the Cdc42-interacting protein 4 (*CIP4*). This protein interacts with the GTPase Cdc42 that is related with actin formation and has been implicated in cytoskeleton organization [40]. Paclitaxel, a type of taxane, promotes microtubule stabilization and polymerization leading to a cell cycle arrest and apoptosis [41]. Actin-microtubule crosstalk is particularly important for cell shape and polarity during cell migration and division [42]. In this context, TRIP10 hypermethylation could increase the efficacy of paclitaxel effects on cytoskeleton. Furthermore, *TRIP10* has an important role in the cellular motility and cohesion control since it is implicated in E-cadherin regulation [43]. In fact, previous studies have related *TRIP10* with cell invasion in TNBC cells in vitro [44]. Interestingly, another study demonstrated also that *TRIP10* gene controls EMT [43]. They described a pro-metastatic role of *TRIP10*, in concordance with the in vitro and in vivo data from Cerqueira et al. [45]. In addition, *TRIP10* expression has shown to be regulated by DNA methylation in mesenchymal stem cell differentiation [46] and in several types of cancer cell lines and tumors [47]. In particular, in breast cancer, it has been also confirmed that *TRIP10* expression can be regulated by epigenetic mechanisms such as DNA methylation [47, 48].

Basing a signature on the methylation status of only two genes could be a limitation of this study. However, the identification of this signature was provided by a very stringent analysis to select the most suitable candidate genes and they were further validated in an independent cohort of patients. In fact, there are several examples of other two-gene signatures, including methylation based signatures, that have previously shown clinical utility for cancer in different types of tumors [49–56]. Additionally, the sample size could be considered small; however, it is important to highlight that the group of patients is very homogeneous in order to eliminate potential confusion factors. In our analysis, we compared methylation levels among TNBC patients who responded and did not respond to neoadjuvant chemotherapy. Both groups were homogeneous also regarding to staging and severity, basing the difference only in the response to chemotherapy. In fact, no statistically significant differences were observed in methylation levels according to staging and severity. Therefore, the strict selection of patients could be a strength that give support and power to these results. The low number of cases analyzed could limit the immediate translational relevance but represents a very good start point for future studies in the field.

## Conclusions

Triple-negative breast cancer has a high relapse rate after conventional chemotherapy treatment. To date, no predictors of treatment effectiveness have been identified. In this study, we propose an epigenetic signature based on the methylation levels of the *FERD3L* and *TRIP10* genes. Our algorithm has a complete pathological response prediction potential of 78.6% and increases the predictive potential described by other approaches. This is especially relevant if we consider that it could be a predictive tool in clinical practice that will allow selecting the appropriate treatment as well as better stratification of patients for clinical trials.

## Methods

### Patients treatment and tumor samples

Patients treated with anthracyclines and/or taxanes NAC in the Hospital Clínico of Valencia and diagnosed with an early TNBC between 2005 and 2015 (Table 1) were retrospectively selected for the study according to clinical inclusion/exclusion criteria (Additional file 7: Table S6). Tumor samples were obtained before exposure to any systemic anticancer treatment using ultrasound-guided core needle biopsy. The cores were placed on OCT and stored at − 80 °C, or included in FFPE. Tumor percentage, histology and ER, PR, HER2, and Ki67 expression were determined. ER and PR status were considered negative when nuclear staining is < 10%. For the assessment of HER2, ASCO/CAP recommendations were used [57]. Diagnosis of TNBC was done according to IHC results. The pathological response after NAC was evaluated by the Symmans method (residual cancer burden; RCB) [19]. A value of RCB = 0 implies pCR, whereas values of RCB > 0 indicates that there is still residual tumor. From an initial analysis of the database, 70 patients were identified. Only those that had a tumor percentage > 25% and reached 500 ng after DNA extraction were used. Fifty-four patients were included in the study; of these, a group of 24 patients (10 RCB = 0 and 14 RCB > 0) were selected for the discovery cohort (DC) and 30 patients (9 RCB = 0 and 21 RCB > 0) were included in the validation cohort (VC).

### Sample size calculation

Sample size calculation was based on data from a recent series reporting that TNBC patients treated with NAC obtaining a RCB = 0 was around 30% [4]. If our methylation data would be able to predict two groups of responders (R) versus non-responders (NR) and we estimate that the proportion of patients with RCB = 0 could be 45% in R while the proportion of RCB = 0 in the NR group would be 10%, with an alpha error of 5% and a power of 80%, the sample size needed to identify this difference between both proportions is 44 patients (22 patients/group). According to this, the theoretical sample size, including a 15% drop-out, should be 51 patients, (https://select-statistics.co.uk/calculators/sample-size-calculator-two-proportions/).

### TNBC cellular lines and treatments

Five TNBC cell lines from American Type Culture Collection (ATCC) were cultured (HCC-1937, HCC-1143, HCC-38, MDA-MB-231, and MDA-MB-436) following standard culture conditions. Treatment with the demethylating agent 5-aza-2′-deoxycytidine (AZA) (Sigma, St. Louis, MO, USA) was performed at 5 uM during 72 h. In this assay, results were performed in triplicates and data were compared with the corresponding non-treated cell line.

### DNA extraction and bisulfite conversion

Both OCT or FFPE TNBC tissue were used depending on availability and DNA extraction was performed using DNA purification protocol with NaCl or the kit “DNA PPPE QIAamp Tissue” (Qiagen) respectively. DNA from cells lines was extracted using Trizol reagent. DNA samples were quantified using PicoGreen method (Invitrogen) and quality was evaluated using Nanodrop (Thermo Scientific) and electrophoresis gels. For DNA bisulfite modification, 500 ng of DNA were used and modification was performed with EZ-96 DNA Methylation (Zymo Research Corp.)

### DNA methylation analysis by Infinium Human Methylation 450 BeadChip array

Microarray-based DNA methylation analysis was conducted with the Infinium Human Methylation 450 BeadChip (450K array; Illumina, San Diego, CA), that covers > 450,000 CpG sites along the human genome [58]. After bisulfite conversion, hybridization was performed following the Illumina Infinium HD methylation protocol.

Methylation score of each CpG was represented as *β* value that ranged between 0 (unmethylated) and 1 (completely methylated). Color balance adjustment and normalization were performed using GenomeStudio Illumina software (V2010.3). After filtering, differentially methylated CpG sites (DMCpGs) between R and NR groups were identified following this flowchart (Fig. 1a): for each probe/CpG, the sets of methylation *β* values belonging to both groups were compared to obtain (1) DMCpGs with a significant *p* value < 0.05 (1030 CpGs); (2) DMCpGs with average *β* values between R and NR groups ≥ 0.20 (133 out of 1030 CpGs); (3) DMCpGs localized in island or shore regions of promotors (35 out of 133 CpGs); (4) DMCpGs with intragroup standard deviation (SD) ≤ 0.20 in order to select the most relevant positions for validation (11 out of 35 CpGs); (5) DMCpGs with a consistent methylation profile in consecutive CpGs (10 out of 11). This final filter with ten CpGs (9 genes) yielded the best candidates for validation.

### DNA methylation analysis by bisulfite pyrosequencing

Quantitative DNA methylation analysis was performed by bisulfite pyrosequencing of consecutive cytosines located in islands or shores of promoter regions of candidate genes using a Pyro Gold SQA™ Reagent Kit (Qiagen) in a PyroMark Q96 System version 2.0.6 (Qiagen) according to the manufacturer’s instructions. CpG site methylation quantification was obtained using Pyro Q-CpG 1.0.9 (Qiagen). Primer sequences (Additional file 8: Table S7) were designed with PyroMark Assay Design 2.0 (Qiagen).

### Gene expression studies and correlation with methylation levels

Total RNA was isolated from OCT/ FFPE samples by mirVana Isolation Kit (Ambion) and from cell lines using Trizol (Invitrogen) according to the manufacturer’s protocol. The RNA (500 ng) were retrotranscribed using the High-Capacity cDNA Reverse Transcription kit (Applied biosystems) according to the manufacturer. Quantitative RT-PCR (qRT-PCR) reactions were performed in triplicate on an Applied Biosystems 7900HT Fast Real-Time PCR system using TaqMan expression assays (FERD3L Hs00541737_s1; TRIP10 Hs00182848_m1; GAPDH Hs03929097_g1 (Applied Biosystems). Gene expression was assayed with GAPDH as endogenous control and using the delta delta Ct method.

### Gene ontology and The Cancer Genome Atlas (TCGA) database analysis

A gene ontology (GO) analysis was performed to estimate the enrichment of the DMCpGs identified in particular biological processes [59]. This analysis detects the significant over-representation of GO terms in one of the sets with respect to the other for the entire genome. GO terms with *p* value < 0.05 were considered significant. DNA methylation and expression data from patients with invasive breast carcinoma were obtained from The Cancer Genome Atlas (TCGA) using the MethHc database (http://methhc.mbc.nctu.edu.tw/php/index.php). Paired DNA methylation and expression data from 713 patients obtained from Infinium 450K array and RNA-Seq, respectively, were used. Based in the methylation results of our study, we considered the methylation data of *FERD3L* promoter region and the methylation of an individual CpG (cg10043037) located at the promoter region.

### Statistical analysis

In the DC, data were summarized by mean, SD, or median. To identify consistent patterns of differentially methylated CpG sites between responders (R) versus non-responders (NR), a non-parametric Wilcoxon rank sum test was performed in the DC. This test demonstrates quite robust results even for a small number of subjects. Globally, a two-tailed *p* value of less than 0.05 was considered to indicate statistical significance. All statistical analyses were performed using GraphPad Prism 7 and R software (version 3.2.0).

In the VC, differences in DNA methylation and transcript levels of the identified genes between R and NR and gene expression changes in the cell lines after the demethylating treatment were assessed by the non-parametric Mann-Whitney *U* test. The correlation between methylation and transcript levels was assessed by Spearman’s rank correlation coefficient.

Receiver operating characteristic (ROC) curves were used to assess the diagnostic predictive capacity of the candidate biomarkers. The area under the curve (AUC) was computed for each ROC curve, and 95% confidence intervals (CI) were also estimated by bootstrapping with 1000 iterations. Sensitivity and specificity were estimated at the optimal cut-off point according to Youden criteria.

Data were summarized by mean, SD, or median. Globally, a two-tailed *p* value of less than 0.05 was considered to indicate statistical significance. All statistical analyses were performed using GraphPad Prism 7 and R software (version 3.2.0).

## Additional files

Additional file 1: List of all the biological processes enriched for the 71 differentially methylated genes between responder and non-responder patients according to the Gene Ontology analysis (DOCX 32 kb)

Additional file 2: Thirty-five differentially methylated CpGs between responders and non-responders group selected from 450k array (delta value  ≥ 0.2) corresponding to 23 genes located in promoter and island/shore (PPT 172 kb)

Additional file 3: Eleven differentially methylated CpGs, corresponding to 11 genes, showed significant methylation differences between non-responder and responder patients: 6 genes (LOC641518; LEF1; HOXA5; EVC2; CDKL2; TLX3) presented a methylation increase in non-responders group vs responders, and 5 genes (ZFHX4; LOC100192378; FERD3L; CHL1; TRIP10) decreased methylation level in non-responder patients compared to those who responded to NAC treatment (PPT 225 kb)

Additional file 4: CpGs studied by pyrosequencing in the DC and in the VC to validate methylation in the candidate genes identified in the 450k array (Illumina). In bold, CpGs from 450k array. Normal type, consecutive CpGs (PPT 140 kb)

Additional file 5: Representation of the pathway interaction network of FERD3L and TRIP10 with other genes using Pathway Commons. FERD3L and TRIP10 are able to interact with different genes that have shown to be implicated in cancer drug resistance (PPT 452 kb)

Additional file 6: Mean differences in methylation levels according to clinicopathological prognostic factors in both cohorts (DC+VC). cT, clinical tumor size; cN, clinical nodule affectation (PPTX 48 kb)

Additional file 7: Clinical inclusion and exclusion criteria followed to select TNBC patients for the methylation study (PPT 89 kb)

Additional file 8: Sequence of primers used by pyrosequencing in the validation assay of candidate genes obtained from 450k array (PPT 143 kb)

## Abbreviations

AIC: Akaike information criterion
ASCO/CAP: American Society of Clinical Oncology/College of American Pathologists
ATCC: American Type Culture Collection
AUC: Area under the curve
AZA: 5-aza-2′-deoxycytidine
bHLH: Basic helix-loop-helix
BL-1: Basal-like 1
BL-2: Basal-like 2
CDKL2: Cyclin-dependent kinase like 2
CHL1: Cell adhesion molecule L1 like
CI: Confidence intervals
CpG: Cytosine and guanine separated by a phosphate
CT: Chemotherapy
Ct: Cycle threshold
DC: Discovery cohort
DMCpGs: Differentially methylated CpG sites
DNA: Deoxyribonucleic acid
E2F4: E2F transcription factor 4
EMT: Epithelial-mesenchymal transition
ER: Estrogen receptors
EVC2: EvC ciliary complex subunit 2
FERD3L: Fer3-like bHLH transcription factor
FFPE: Formalin-fixed paraffin-embedded
GAPDH: Glyceraldehyde-3-phosphate dehydrogenase
GO: Gene ontology
HER2: Erb-b2 receptor tyrosine kinase 2
HOXA5: Homeobox A5
IHC: Immunohistochemistry
LAR: Luminal androgen receptor
LEF1: Lymphoid enhancer binding factor 1
M: Mesenchymal
MethHc: A database of DNA Methylation and gene expression in Human Cancer
NAC: Neoadjuvant chemotherapy
Non-pCR: Non-pathological complete response
NR: Non-responders
pCR: Pathological complete response
PR: Progesterone receptor
qRT-PCR: Quantitative reverse transcriptase polymerase chain reaction
R: Responders
RCB: Residual cancer burden
RNA-seq: RNA sequencing (RNA: ribonucleic acid)
ROC: Receiver operating characteristic
RT-PCR: Reverse transcriptase polymerase chain reaction
SD: Standard deviation
siRNA: Small interfering RNA
TCGA: The cancer genome atlas
TLX3: T cell leukemia homeobox 3
TN: Triple negative
TNBC: Triple-negative breast cancer
TRIP10/CIP4: Thyroid hormone receptor interactor 10
TWIST: Twist family bHLH transcription factor 1
VC: Validation cohort
