# Supplementary material for: A two-gene epigenetic signature for the prediction of response to neoadjuvant chemotherapy in triple-negative breast cancer patients
Source: Clin Epigenetics. 2019 Feb 20;11:33. doi: 10.1186/s13148-019-0626-0 (PMC6381754; doi:10.1186/s13148-019-0626-0)
Supplement: Supplementary file 1 — List of all the biological processes enriched for the 71 differentially methylated genes between responder and non-responder patients according to the Gene Ontology analysis (DOCX 32 kb) [file 13148_2019_626_MOESM1_ESM.docx]

**Table S1:**  List of all the biological processes enriched for the 71 differentially methylated genes between responder and non-responder patients according to the Gene Ontology analysis.

| **GO term** | **Biological process** | **p-value** | **Adjusted p-value (FDR)** | **Genes** |
| --- | --- | --- | --- | --- |
| GO:0007155 | cell adhesion | 0.000001 | 0.000678 | **LEF1**,NRXN3,CNTNAP2,NID2,  PCDH15,NTM,**CHL1**,LSAMP,CD96 |
| GO:0007275 | multicellular organismal development | 0.000562 | 0.020393 | ZIC2,INSC,EBF1,PAX9,PAX7,**CHL1**,  EYA4,**TLX3** |
| GO:0045892 | negative regulation of transcription, DNA-dependent | 0.000148 | 0.010745 | **LEF1**,HDAC9,ZIC2,PAX9,BMP4,**FERD3L** |
| GO:0007399 | nervous system development | 0.001354 | 0.034660 | INSC,PCSK2,IGSF8,**CHL1**,LSAMP |
| GO:0050896 | response to stimulus | 0.004536 | 0.022947 | OR2T6,OR2M4,OR51B2,OR2M3,OR6K6 |
| GO:0000122 | negative regulation of transcription from RNA polymerase II promoter | 0.009522 | 0.032616 | **LEF1**,HDAC9,BMP4,DNAJA3 |
| GO:0008285 | negative regulation of cell proliferation | 0.004786 | 0.023660 | PTH1R,BMP4,DLC1,DNAJA3 |
| GO:0045893 | positive regulation of transcription, DNA-dependent | 0.014154 | 0.036218 | **LEF1**,ZIC2,EBF1,BMP4 |
| GO:0007601 | visual perception | 0.000445 | 0.017604 | ZIC2,POU6F2,PCDH15,EYA4 |
| GO:0043065 | positive regulation of apoptotic process | 0.005418 | 0.025899 | BMP4,DNAJA3,**HOXA5** |
| GO:0007264 | small GTPase mediated signal transduction | 0.024364 | 0.048174 | DLC1,DNAJA3,**TRIP10** |
| GO:0006281 | DNA repair | 0.019264 | 0.042975 | SETMAR,MGMT,EYA4 |
| GO:0007411 | axon guidance | 0.023369 | 0.048179 | NRXN3,KCNQ3,**CHL1** |
| GO:0043066 | negative regulation of apoptotic process | 0.017045 | 0.040964 | **LEF1**,BMP4,DNAJA3 |
| GO:0016568 | chromatin modification | 0.010157 | 0.031115 | SETMAR,HDAC9,EYA4 |
| GO:0007420 | brain development | 0.005502 | 0.026017 | **LEF1**,ZIC2,CNTNAP2 |
| GO:0009653 | anatomical structure morphogenesis | 0.015435 | 0.039265 | PAX7,EYA4 |
| GO:0031175 | neuron projection development | 0.00792 | 0.030490 | CNTNAP2,**CHL1** |
| GO:0001822 | kidney development | 0.010093 | 0.031138 | **LEF1**,BMP4 |
| GO:0032869 | cellular response to insulin stimulus | 0.005594 | 0.026169 | HDAC9,DLC1 |
| GO:0002062 | chondrocyte differentiation | 0.001323 | 0.035980 | PTH1R,BMP4 |
| GO:0001649 | osteoblast differentiation | 0.003335 | 0.027377 | **LEF1**,BMP4 |
| GO:0007519 | skeletal muscle tissue development | 0.00414 | 0.021187 | PAX7,IGSF8 |
| GO:0008203 | cholesterol metabolic process | 0.006192 | 0.024050 | CUBN,CYP7B1 |
| GO:0007608 | sensory perception of smell | 0.005212 | 0.025192 | OR2M4,OR51B2 |
| GO:0007610 | behavior | 0.001975 | 0.042967 | CNTNAP2,**CHL1** |
| GO:0001764 | neuron migration | 0.010608 | 0.032269 | **CHL1**,**TLX3** |
| GO:0060548 | negative regulation of cell death | 0.001134 | 0.035236 | BMP4,MGMT |
| GO:0007224 | smoothened signaling pathway | 0.001858 | 0.044905 | BMP4,**EVC2** |
| GO:0007417 | central nervous system development | 0.021029 | 0.045063 | POU6F2,**TLX3** |
| GO:0030509 | BMP signaling pathway | 0.003647 | 0.029383 | **LEF1**,BMP4 |
| GO:0050953 | sensory perception of light stimulus | 0.000138 | 0.012085 | PCDH15,USH1C |
| GO:0007605 | sensory perception of sound | 0.018971 | 0.042538 | PCDH15,USH1C |
| GO:0050957 | equilibrioception | 0.000058 | 0.006318 | PCDH15,USH1C |
| GO:0045494 | photoreceptor cell maintenance | 0.001134 | 0.035236 | PCDH15,USH1C |
| GO:0042491 | auditory receptor cell differentiation | 0.000173 | 0.010776 | PCDH15,USH1C |
| GO:0048286 | lung alveolus development | 0.001975 | 0.042967 | BMP4,**HOXA5** |
| GO:0001843 | neural tube closure | 0.004661 | 0.023308 | ZIC2,DLC1 |
| GO:0050911 | detection of chemical stimulus involved in sensory perception of smell | 0.002096 | 0.017880 | OR2M4,OR51B2 |
| GO:0006919 | activation of cysteine-type endopeptidase activity involved in apoptotic process | 0.008856 | 0.030577 | DLC1,DNAJA3 |
| GO:0002009 | morphogenesis of an epithelium | 0.000402 | 0.017488 | PCDH15,**HOXA5** |
| GO:0060644 | mammary gland epithelial cell differentiation | 0.000299 | 0.014472 | MGMT,**HOXA5** |
| GO:0060441 | epithelial tube branching involved in lung morphogenesis | 0.000299 | 0.014472 | BMP4,**HOXA5** |
| GO:0035264 | multicellular organism growth | 0.003335 | 0.027377 | PCDH15,**HOXA5** |
| GO:0043154 | negative regulation of cysteine-type endopeptidase activity involved in apoptotic process | 0.005025 | 0.024562 | **LEF1**,DNAJA3 |
| GO:0060325 | face morphogenesis | 0.001226 | 0.035581 | **LEF1**,PAX9 |
| GO:0008038 | neuron recognition | 0.000038 | 0.005623 | CNTNAP2,NTM |
| GO:0045843 | negative regulation of striated muscle tissue development | 0.000038 | 0.005623 | **LEF1**,BMP4 |
| GO:0045662 | negative regulation of myoblast differentiation | 0.017751 | 0.040008 | BMP4 |
| GO:0090184 | positive regulation of kidney development | 0.021653 | 0.044853 | BMP4 |
| GO:0045639 | positive regulation of myeloid cell differentiation | 0.0099 | 0.030761 | **HOXA5** |
| GO:0071353 | cellular response to interleukin-4 | 0.007928 | 0.027589 | **LEF1** |
| GO:0010226 | response to lithium ion | 0.021653 | 0.044853 | **LEF1** |
| GO:0071864 | positive regulation of cell proliferation in bone marrow | 0.003971 | 0.020568 | **LEF1** |
| GO:0003139 | secondary heart field specification | 0.0099 | 0.030762 | BMP4 |
| GO:0042100 | B cell proliferation | 0.02554 | 0.049599 | **LEF1** |
| GO:0060425 | lung morphogenesis | 0.017751 | 0.040009 | BMP4 |
| GO:0021527 | spinal cord association neuron differentiation | 0.023598 | 0.046874 | PAX7 |
| GO:0030854 | positive regulation of granulocyte differentiation | 0.0099 | 0.030762 | **LEF1** |
| GO:0035307 | positive regulation of protein dephosphorylation | 0.015794 | 0.038169 | DLC1 |
| GO:0030046 | parallel actin filament bundle assembly | 0.003971 | 0.020568 | USH1C |
| GO:0021756 | striatum development | 0.013833 | 0.035607 | CNTNAP2 |
| GO:0006924 | activation-induced cell death of T cells | 0.011868 | 0.033309 | DNAJA3 |
| GO:0030225 | macrophage differentiation | 0.02554 | 0.049599 | BMP4 |
| GO:0071625 | vocalization behavior | 0.0099 | 0.030762 | CNTNAP2 |
| GO:0060764 | cell-cell signaling involved in mammary gland development | 0.001987 | 0.017294 | **HOXA5** |
| GO:0060435 | bronchiole development | 0.003971 | 0.020568 | **HOXA5** |
| GO:0033599 | regulation of mammary gland epithelial cell proliferation | 0.005951 | 0.023325 | **HOXA5** |
| GO:0060535 | trachea cartilage morphogenesis | 0.007928 | 0.027590 | **HOXA5** |
| GO:0060480 | lung goblet cell differentiation | 0.003971 | 0.020568 | **HOXA5** |
| GO:0010870 | positive regulation of receptor biosynthetic process | 0.013833 | 0.035607 | **HOXA5** |
| GO:0003016 | respiratory system process | 0.013833 | 0.035607 | **HOXA5** |
| GO:0060638 | mesenchymal-epithelial cell signaling | 0.005951 | 0.023325 | **HOXA5** |
| GO:0060484 | lung-associated mesenchyme development | 0.017751 | 0.040009 | **HOXA5** |
| GO:0045647 | negative regulation of erythrocyte differentiation | 0.015794 | 0.038169 | **HOXA5** |
| GO:0060574 | intestinal epithelial cell maturation | 0.003971 | 0.020568 | **HOXA5** |
| GO:0048699 | generation of neurons | 0.02554 | 0.049599 | **LEF1** |
| GO:0060438 | trachea development | 0.005951 | 0.023325 | BMP4 |
| GO:0072001 | renal system development | 0.011868 | 0.033309 | BMP4 |
| GO:0003197 | endocardial cushion development | 0.013833 | 0.035607 | BMP4 |
| GO:0016202 | regulation of striated muscle tissue development | 0.0099 | 0.030762 | **LEF1** |
| GO:0000132 | establishment of mitotic spindle orientation | 0.02554 | 0.049599 | INSC |
| GO:0060391 | positive regulation of SMAD protein import into nucleus | 0.015794 | 0.038169 | BMP4 |
| GO:0060033 | anatomical structure regression | 0.005951 | 0.023325 | **LEF1** |
| GO:0006266 | DNA ligation | 0.013833 | 0.035607 | MGMT |
| GO:0033153 | T cell receptor V(D)J recombination | 0.011868 | 0.033309 | **LEF1** |
| GO:0051298 | centrosome duplication | 0.019704 | 0.042432 | ARHGEF10 |
| GO:0006264 | mitochondrial DNA replication | 0.015794 | 0.038169 | DNAJA3 |
| GO:0045778 | positive regulation of ossification | 0.023598 | 0.046874 | BMP4 |
| GO:0002040 | sprouting angiogenesis | 0.023598 | 0.046874 | **LEF1** |
| GO:0002087 | regulation of respiratory gaseous exchange by neurological system process | 0.019704 | 0.042432 | **TLX3** |
| GO:0022408 | negative regulation of cell-cell adhesion | 0.021653 | 0.044853 | **LEF1** |
| GO:0030111 | regulation of Wnt receptor signaling pathway | 0.023598 | 0.046874 | **LEF1** |
| GO:0042481 | regulation of odontogenesis | 0.003971 | 0.020568 | PAX9 |
| GO:0051497 | negative regulation of stress fiber assembly | 0.0099 | 0.030762 | DLC1 |
| GO:0042359 | vitamin D metabolic process | 0.019704 | 0.042432 | CUBN |
| GO:0050910 | detection of mechanical stimulus involved in sensory perception of sound | 0.015794 | 0.038169 | PCDH15 |
| GO:0021854 | hypothalamus development | 0.023598 | 0.046874 | **LEF1** |
| GO:0090026 | positive regulation of monocyte chemotaxis | 0.013833 | 0.035607 | S100A14 |
| GO:0042953 | lipoprotein transport | 0.021653 | 0.044853 | CUBN |
| GO:0048066 | developmental pigmentation | 0.023598 | 0.046874 | ZIC2 |
| GO:0022407 | regulation of cell-cell adhesion | 0.017751 | 0.040009 | **LEF1** |
| GO:0006002 | fructose 6-phosphate metabolic process | 0.013833 | 0.035607 | PFKP |
| GO:0030388 | fructose 1,6-bisphosphate metabolic process | 0.011868 | 0.033309 | PFKP |
| GO:0048665 | neuron fate specification | 0.017751 | 0.040009 | **TLX3** |
| GO:0071899 | negative regulation of estrogen receptor binding | 0.003971 | 0.020568 | **LEF1** |
| GO:0060336 | negative regulation of interferon-gamma-mediated signaling pathway | 0.007928 | 0.027590 | DNAJA3 |
| GO:0043923 | positive regulation by host of viral transcription | 0.019704 | 0.042432 | **LEF1** |
| GO:0071157 | negative regulation of cell cycle arrest | 0.017751 | 0.040009 | SETMAR |
| GO:0021904 | dorsal/ventral neural tube patterning | 0.021653 | 0.044853 | PAX7 |
| GO:0002320 | lymphoid progenitor cell differentiation | 0.007928 | 0.027590 | BMP4 |
| GO:0071624 | positive regulation of granulocyte chemotaxis | 0.003971 | 0.020568 | S100A14 |
| GO:0002244 | hemopoietic progenitor cell differentiation | 0.019704 | 0.042432 | BMP4 |
| GO:0021542 | dentate gyrus development | 0.019704 | 0.042432 | **LEF1** |
| GO:0015074 | DNA integration | 0.023598 | 0.046874 | SETMAR |
| GO:0021794 | thalamus development | 0.015794 | 0.038169 | CNTNAP2 |
| GO:0060740 | prostate gland epithelium morphogenesis | 0.017751 | 0.040009 | CYP7B1 |
| GO:0003337 | mesenchymal to epithelial transition involved in metanephros morphogenesis | 0.013833 | 0.035607 | BMP4 |
| GO:0002043 | blood vessel endothelial cell proliferation involved in sprouting angiogenesis | 0.007928 | 0.027590 | BMP4 |
| GO:0060393 | regulation of pathway-restricted SMAD protein phosphorylation | 0.005951 | 0.023325 | BMP4 |
| GO:0043586 | tongue development | 0.015794 | 0.038169 | **LEF1** |
| GO:0035024 | negative regulation of Rho protein signal transduction | 0.015794 | 0.038169 | DLC1 |
| GO:0051593 | response to folic acid | 0.017751 | 0.040009 | MGMT |
| GO:0035058 | nonmotile primary cilium assembly | 0.013833 | 0.035607 | PCDH15 |
| GO:0043306 | positive regulation of mast cell degranulation | 0.017751 | 0.040009 | FCER1A |
| GO:0033085 | negative regulation of T cell differentiation in thymus | 0.0099 | 0.030762 | BMP4 |
| GO:0033088 | negative regulation of immature T cell proliferation in thymus | 0.0099 | 0.030762 | BMP4 |
| GO:0046135 | pyrimidine nucleoside catabolic process | 0.023598 | 0.046874 | NT5C3 |
| GO:0061036 | positive regulation of cartilage development | 0.021653 | 0.044853 | BMP4 |
| GO:0045839 | negative regulation of mitosis | 0.011868 | 0.033309 | BMP4 |
| GO:0072125 | negative regulation of glomerular mesangial cell proliferation | 0.003971 | 0.020568 | BMP4 |
| GO:0070933 | histone H4 deacetylation | 0.011868 | 0.033309 | HDAC9 |
| GO:0045163 | clustering of voltage-gated potassium channels | 0.005951 | 0.023325 | CNTNAP2 |
| GO:0035993 | deltoid tuberosity development | 0.005951 | 0.023325 | BMP4 |
| GO:0035990 | tendon cell differentiation | 0.003971 | 0.020568 | BMP4 |
| GO:0060415 | muscle tissue morphogenesis | 0.007928 | 0.027590 | PAX7 |
| GO:0043403 | skeletal muscle tissue regeneration | 0.017751 | 0.040009 | PAX7 |
| GO:0030070 | insulin processing | 0.003971 | 0.020568 | PCSK2 |
| GO:0021575 | hindbrain morphogenesis | 0.005951 | 0.023325 | DLC1 |
| GO:0046632 | alpha-beta T cell differentiation | 0.011868 | 0.033309 | **LEF1** |
| GO:0032713 | negative regulation of interleukin-4 production | 0.0099 | 0.030762 | **LEF1** |
| GO:0046085 | adenosine metabolic process | 0.007928 | 0.027590 | NT5C3 |
| GO:0022011 | myelination in peripheral nervous system | 0.013833 | 0.035607 | ARHGEF10 |
| GO:0015889 | cobalamin transport | 0.0099 | 0.030762 | CUBN |
| GO:0071109 | superior temporal gyrus development | 0.003971 | 0.020568 | CNTNAP2 |
| GO:0032714 | negative regulation of interleukin-5 production | 0.007928 | 0.027590 | **LEF1** |
| GO:0032696 | negative regulation of interleukin-13 production | 0.005951 | 0.023325 | **LEF1** |
| GO:0022409 | positive regulation of cell-cell adhesion | 0.021653 | 0.044853 | **LEF1** |
| GO:0034983 | peptidyl-lysine deacetylation | 0.007928 | 0.027590 | HDAC9 |
| GO:0021873 | forebrain neuroblast division | 0.003971 | 0.020568 | **LEF1** |
| GO:0070244 | negative regulation of thymocyte apoptosis | 0.0099 | 0.030762 | BMP4 |
| GO:0030223 | neutrophil differentiation | 0.003971 | 0.020568 | **LEF1** |
| GO:0090307 | spindle assembly involved in mitosis | 0.015794 | 0.038169 | ARHGEF10 |
| GO:0006307 | DNA dealkylation involved in DNA repair | 0.011868 | 0.033309 | MGMT |
| GO:0001812 | positive regulation of type I hypersensitivity | 0.003971 | 0.020568 | FCER1A |
| GO:0001964 | startle response | 0.011868 | 0.033309 | PCDH15 |
| GO:0060013 | righting reflex | 0.013833 | 0.035607 | PCDH15 |
| GO:0072193 | ureter smooth muscle cell differentiation | 0.003971 | 0.020568 | BMP4 |
| GO:0060088 | auditory receptor cell stereocilium organization | 0.013833 | 0.035607 | PCDH15 |
| GO:0034230 | enkephalin processing | 0.001987 | 0.017294 | PCSK2 |
| GO:0034231 | islet amyloid polypeptide processing | 0.001987 | 0.017294 | PCSK2 |
| GO:0016540 | protein autoprocessing | 0.017751 | 0.040009 | PCSK2 |
| GO:0072205 | metanephric collecting duct development | 0.011868 | 0.033309 | BMP4 |
| GO:0021879 | forebrain neuron differentiation | 0.013833 | 0.035607 | **LEF1** |
| GO:0072104 | glomerular capillary formation | 0.003971 | 0.020568 | BMP4 |
| GO:0090050 | positive regulation of cell migration involved in sprouting angiogenesis | 0.011868 | 0.033309 | HDAC9 |
| GO:0060487 | lung epithelial cell differentiation | 0.017751 | 0.040009 | INSC |
| GO:0007402 | ganglion mother cell fate determination | 0.003971 | 0.020568 | POU6F2 |
| GO:0060561 | apoptosis involved in morphogenesis | 0.0099 | 0.030762 | **LEF1** |
| GO:0048747 | muscle fiber development | 0.011868 | 0.033309 | **LEF1** |
| GO:0048341 | paraxial mesoderm formation | 0.007928 | 0.027590 | **LEF1** |
| GO:0043281 | regulation of cysteine-type endopeptidase activity involved in apoptotic process | 0.013833 | 0.035607 | MGMT |
| GO:0021861 | forebrain radial glial cell differentiation | 0.007928 | 0.027590 | **LEF1** |
| GO:0021943 | formation of radial glial scaffolds | 0.003971 | 0.020568 | **LEF1** |
| GO:0019226 | transmission of nerve impulse | 0.019704 | 0.042432 | CNTNAP2 |
| GO:0034389 | lipid particle organization | 0.005951 | 0.023325 | PTGFRN |
| GO:0050973 | detection of mechanical stimulus involved in equilibrioception | 0.001987 | 0.017294 | PCDH15 |
| GO:0070932 | histone H3 deacetylation | 0.007928 | 0.027590 | HDAC9 |
| GO:0045401 | positive regulation of interleukin-3 biosynthetic process | 0.003971 | 0.020568 | FCER1A |
| GO:0045425 | positive regulation of granulocyte macrophage colony-stimulating factor biosynthetic process | 0.005951 | 0.023325 | FCER1A |
| GO:0001820 | serotonin secretion | 0.003971 | 0.020568 | FCER1A |
| GO:0006213 | pyrimidine nucleoside metabolic process | 0.005951 | 0.023325 | NT5C3 |
| GO:0060710 | chorio-allantoic fusion | 0.0099 | 0.030762 | **LEF1** |
| GO:0033147 | negative regulation of intracellular estrogen receptor signaling pathway | 0.005951 | 0.023325 | CYP7B1 |
| GO:0061047 | positive regulation of branching involved in lung morphogenesis | 0.003971 | 0.020568 | BMP4 |
| GO:0000729 | DNA double-strand break processing | 0.005951 | 0.023325 | SETMAR |
| GO:0048742 | regulation of skeletal muscle fiber development | 0.003971 | 0.020568 | HDAC9 |
| GO:0071205 | protein localization to juxtaparanode region of axon | 0.003971 | 0.020568 | CNTNAP2 |
| GO:0021761 | limbic system development | 0.003971 | 0.020568 | CNTNAP2 |
| GO:0071340 | skeletal muscle acetylcholine-gated channel clustering | 0.005951 | 0.023325 | DNAJA3 |
| GO:0048069 | eye pigmentation | 0.003971 | 0.020568 | **LEF1** |
| GO:0033126 | positive regulation of GTP catabolic process | 0.003971 | 0.020568 | ARHGEF10 |
| GO:0014813 | satellite cell commitment | 0.001987 | 0.017294 | PAX7 |
| GO:0061153 | trachea gland development | 0.005951 | 0.023325 | **LEF1** |
| GO:0003130 | BMP signaling pathway involved in heart induction | 0.005951 | 0.023325 | BMP4 |
| GO:0072097 | negative regulation of branch elongation involved in ureteric bud branching by BMP signaling pathway | 0.001987 | 0.017294 | BMP4 |
| GO:0090191 | negative regulation of branching involved in ureteric bud morphogenesis | 0.001987 | 0.017294 | BMP4 |
| GO:2000137 | negative regulation of cell proliferation involved in heart morphogenesis | 0.001987 | 0.017294 | BMP4 |
| GO:0060433 | bronchus development | 0.001987 | 0.017294 | BMP4 |
| GO:0003279 | cardiac septum development | 0.005951 | 0.023325 | BMP4 |
| GO:0090194 | negative regulation of glomerulus development | 0.001987 | 0.017294 | BMP4 |
| GO:0061149 | BMP signaling pathway involved in ureter morphogenesis | 0.001987 | 0.017294 | BMP4 |
| GO:0072200 | negative regulation of mesenchymal cell proliferation involved in ureter development | 0.001987 | 0.017294 | BMP4 |
| GO:0072101 | specification of ureteric bud anterior/posterior symmetry by BMP signaling pathway | 0.001987 | 0.017294 | BMP4 |
| GO:0061151 | BMP signaling pathway involved in renal system segmentation | 0.001987 | 0.017294 | BMP4 |
| GO:0061155 | pulmonary artery endothelial tube morphogenesis | 0.001987 | 0.017294 | BMP4 |
| GO:2000005 | negative regulation of metanephric S-shaped body morphogenesis | 0.001987 | 0.017294 | BMP4 |
| GO:0072192 | ureter epithelial cell differentiation | 0.001987 | 0.017294 | BMP4 |
| GO:0071893 | BMP signaling pathway involved in nephric duct formation | 0.001987 | 0.017294 | BMP4 |
| GO:0055020 | positive regulation of cardiac muscle fiber development | 0.001987 | 0.017294 | BMP4 |
| GO:2000007 | negative regulation of metanephric comma-shaped body morphogenesis | 0.001987 | 0.017294 | BMP4 |
| GO:0048392 | intermediate mesodermal cell differentiation | 0.001987 | 0.017294 | BMP4 |
| GO:0060502 | epithelial cell proliferation involved in lung morphogenesis | 0.001987 | 0.017294 | BMP4 |
| GO:0060503 | bud dilation involved in lung branching | 0.001987 | 0.017294 | BMP4 |
| GO:0090068 | positive regulation of cell cycle process | 0.003971 | 0.020568 | **LEF1** |
| GO:0045063 | T-helper 1 cell differentiation | 0.003971 | 0.020568 | **LEF1** |
| GO:0020028 | hemoglobin import | 0.001987 | 0.017294 | CUBN |
| GO:0006313 | transposition, DNA-mediated | 0.003971 | 0.020568 | SETMAR |
| GO:0051153 | regulation of striated muscle cell differentiation | 0.001987 | 0.017294 | HDAC9 |
| GO:0071866 | negative regulation of apoptotic process in bone marrow | 0.001987 | 0.017294 | **LEF1** |
| GO:0071895 | odontoblast differentiation | 0.001987 | 0.017294 | **LEF1** |
| GO:2001251 | negative regulation of chromosome organization | 0.001987 | 0.017294 | SETMAR |
| GO:2001034 | positive regulation of double-strand break repair via nonhomologous end joining | 0.001987 | 0.017294 | SETMAR |
| GO:0000737 | DNA catabolic process, endonucleolytic | 0.001987 | 0.017294 | SETMAR |

Genes in bold represent candidate genes with significant methylation differences between responder and non-responder patients that were analyzed for validation by pyrosequencing analysis.
