# Supplementary material for: A two-gene epigenetic signature for the prediction of response to neoadjuvant chemotherapy in triple-negative breast cancer patients
Source: Clin Epigenetics. 2019 Feb 20;11:33. doi: 10.1186/s13148-019-0626-0 (PMC6381754; doi:10.1186/s13148-019-0626-0)
Supplement: Supplementary file 2 — Thirty-five differentially methylated CpGs between responders and non-responders group selected from 450k array (delta value ≥ 0.2) corresponding to 23 genes located in promoter and island/shore (PPT 172 kb) [file 13148_2019_626_MOESM2_ESM.ppt]

## Slide 1
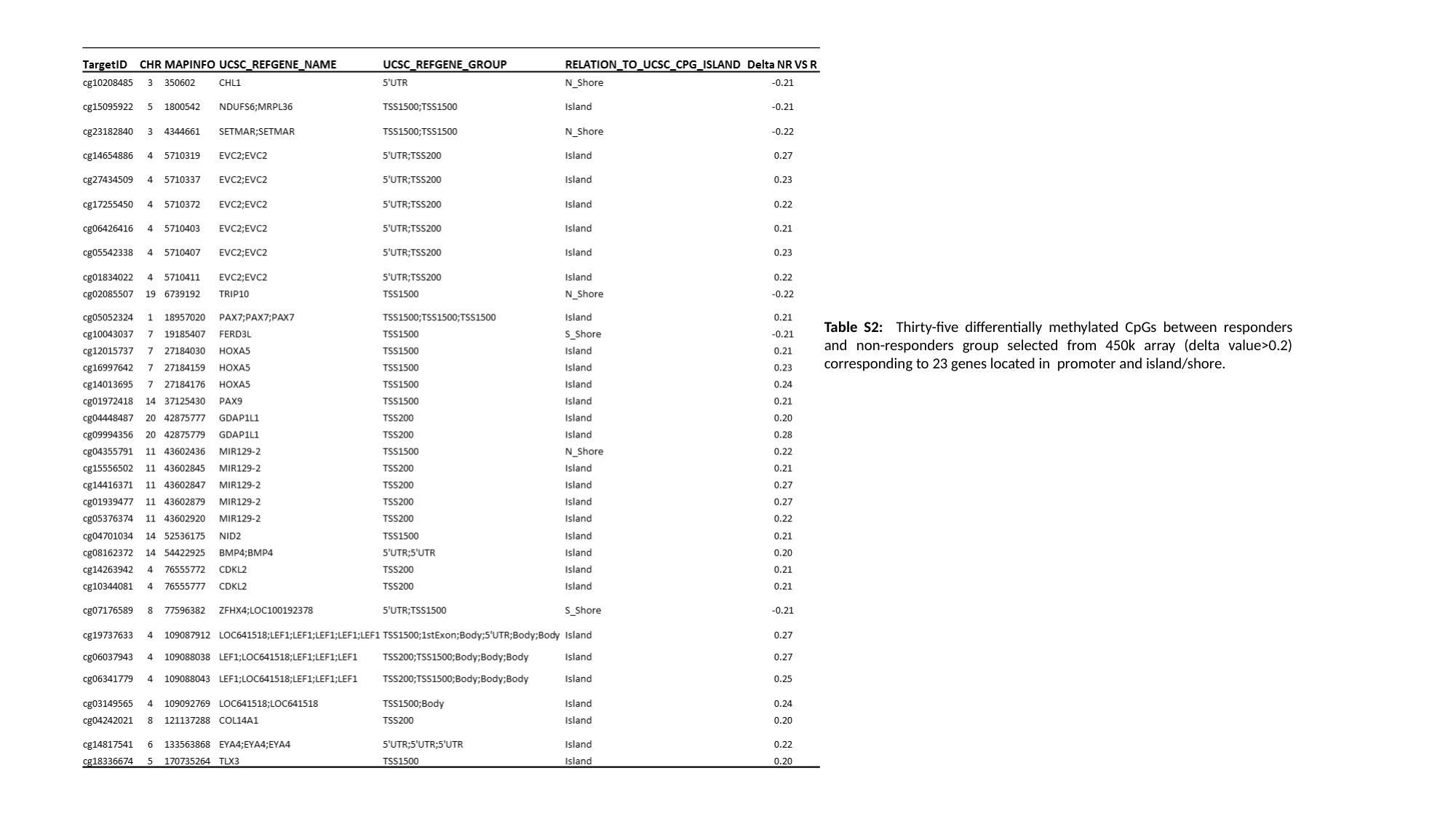

Table S2: Thirty-five differentially methylated CpGs between responders and non-responders group selected from 450k array (delta value>0.2) corresponding to 23 genes located in promoter and island/shore.
