# Supplementary material for: A two-gene epigenetic signature for the prediction of response to neoadjuvant chemotherapy in triple-negative breast cancer patients
Source: Clin Epigenetics. 2019 Feb 20;11:33. doi: 10.1186/s13148-019-0626-0 (PMC6381754; doi:10.1186/s13148-019-0626-0)
Supplement: Supplementary file 3 — Eleven differentially methylated CpGs, corresponding to 11 genes, showed significant methylation differences between non-responder and responder patients: 6 genes (LOC641518; LEF1; HOXA5; EVC2; CDKL2; TLX3) presented a methylation increase in non-responders group vs responders, and 5 genes (ZFHX4; LOC100192378; FERD3L; CHL1; TRIP10) decreased methylation level in non-responder patients compared to those who responded to NAC treatment (PPT 225 kb) [file 13148_2019_626_MOESM3_ESM.ppt]

## Slide 1
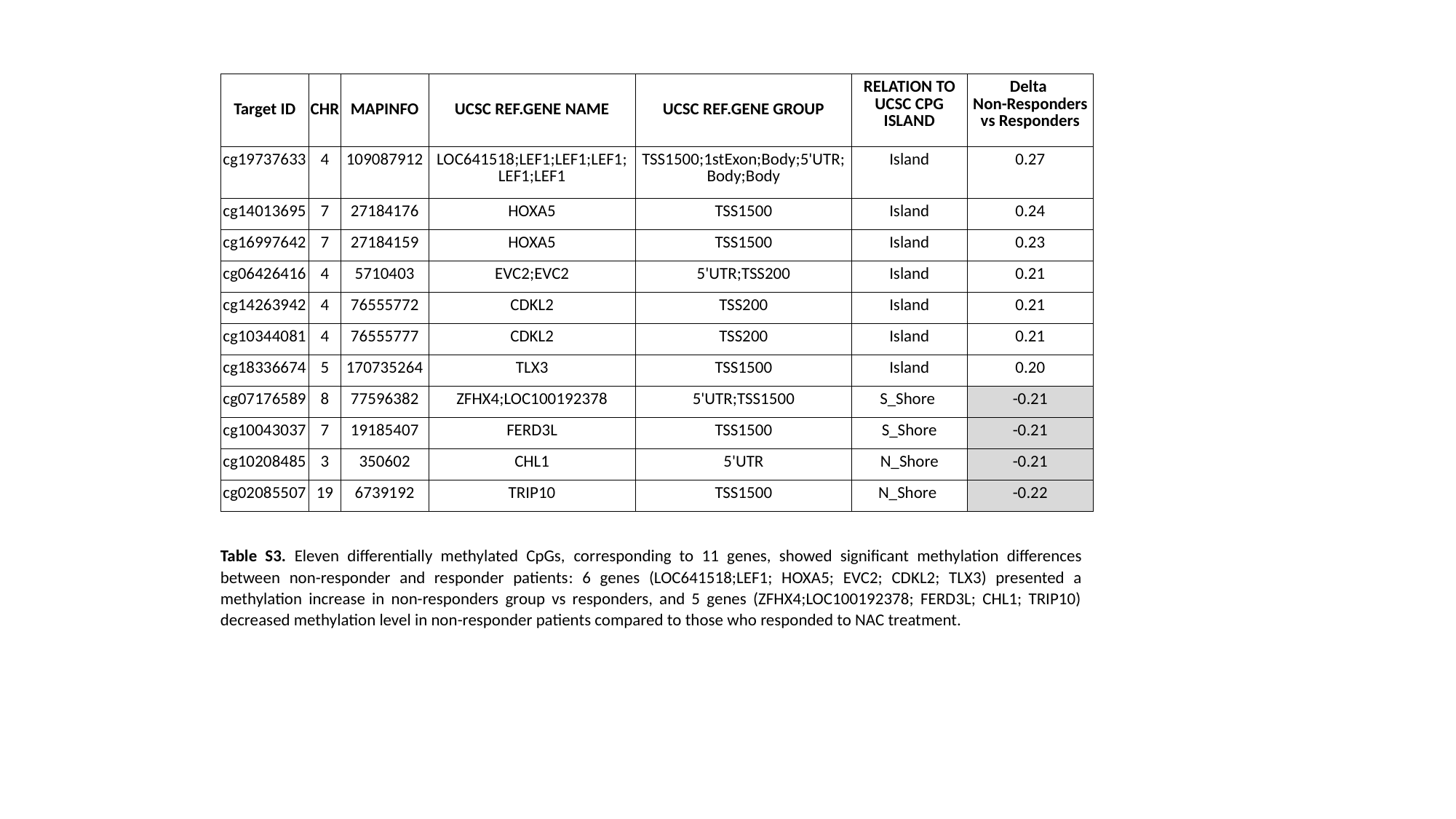

| Target ID | CHR | MAPINFO | UCSC REF.GENE NAME | UCSC REF.GENE GROUP | RELATION TO UCSC CPG ISLAND | Delta Non-Responders vs Responders |
| --- | --- | --- | --- | --- | --- | --- |
| cg19737633 | 4 | 109087912 | LOC641518;LEF1;LEF1;LEF1; LEF1;LEF1 | TSS1500;1stExon;Body;5'UTR; Body;Body | Island | 0.27 |
| cg14013695 | 7 | 27184176 | HOXA5 | TSS1500 | Island | 0.24 |
| cg16997642 | 7 | 27184159 | HOXA5 | TSS1500 | Island | 0.23 |
| cg06426416 | 4 | 5710403 | EVC2;EVC2 | 5'UTR;TSS200 | Island | 0.21 |
| cg14263942 | 4 | 76555772 | CDKL2 | TSS200 | Island | 0.21 |
| cg10344081 | 4 | 76555777 | CDKL2 | TSS200 | Island | 0.21 |
| cg18336674 | 5 | 170735264 | TLX3 | TSS1500 | Island | 0.20 |
| cg07176589 | 8 | 77596382 | ZFHX4;LOC100192378 | 5'UTR;TSS1500 | S\_Shore | -0.21 |
| cg10043037 | 7 | 19185407 | FERD3L | TSS1500 | S\_Shore | -0.21 |
| cg10208485 | 3 | 350602 | CHL1 | 5'UTR | N\_Shore | -0.21 |
| cg02085507 | 19 | 6739192 | TRIP10 | TSS1500 | N\_Shore | -0.22 |
Table S3. Eleven differentially methylated CpGs, corresponding to 11 genes, showed significant methylation differences between non-responder and responder patients: 6 genes (LOC641518;LEF1; HOXA5; EVC2; CDKL2; TLX3) presented a methylation increase in non-responders group vs responders, and 5 genes (ZFHX4;LOC100192378; FERD3L; CHL1; TRIP10) decreased methylation level in non-responder patients compared to those who responded to NAC treatment.
