# Supplementary material for: A two-gene epigenetic signature for the prediction of response to neoadjuvant chemotherapy in triple-negative breast cancer patients
Source: Clin Epigenetics. 2019 Feb 20;11:33. doi: 10.1186/s13148-019-0626-0 (PMC6381754; doi:10.1186/s13148-019-0626-0)
Supplement: Supplementary file 4 — CpGs studied by pyrosequencing in the DC and in the VC to validate methylation in the candidate genes identified in the 450k array (Illumina). In bold, CpGs from 450k array. Normal type, consecutive CpGs (PPT 140 kb) [file 13148_2019_626_MOESM4_ESM.ppt]

## Slide 1
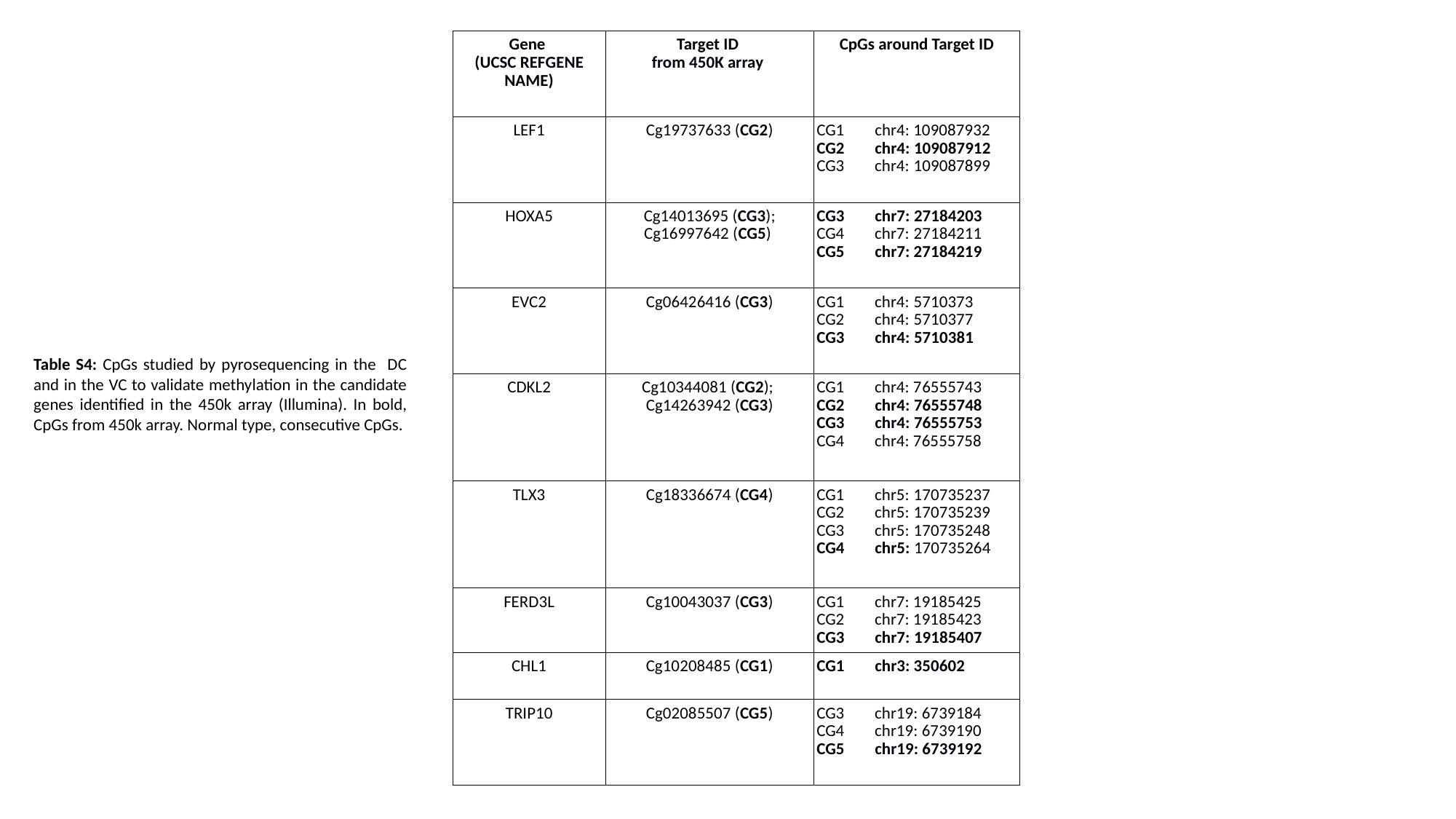

| Gene (UCSC REFGENE NAME) | Target ID from 450K array | CpGs around Target ID |
| --- | --- | --- |
| LEF1 | Cg19737633 (CG2) | CG1 chr4: 109087932 CG2 chr4: 109087912 CG3 chr4: 109087899 |
| HOXA5 | Cg14013695 (CG3); Cg16997642 (CG5) | CG3 chr7: 27184203 CG4 chr7: 27184211 CG5 chr7: 27184219 |
| EVC2 | Cg06426416 (CG3) | CG1 chr4: 5710373 CG2 chr4: 5710377 CG3 chr4: 5710381 |
| CDKL2 | Cg10344081 (CG2); Cg14263942 (CG3) | CG1 chr4: 76555743 CG2 chr4: 76555748 CG3 chr4: 76555753 CG4 chr4: 76555758 |
| TLX3 | Cg18336674 (CG4) | CG1 chr5: 170735237 CG2 chr5: 170735239 CG3 chr5: 170735248 CG4 chr5: 170735264 |
| FERD3L | Cg10043037 (CG3) | CG1 chr7: 19185425 CG2 chr7: 19185423 CG3 chr7: 19185407 |
| CHL1 | Cg10208485 (CG1) | CG1 chr3: 350602 |
| TRIP10 | Cg02085507 (CG5) | CG3 chr19: 6739184 CG4 chr19: 6739190 CG5 chr19: 6739192 |
Table S4: CpGs studied by pyrosequencing in the DC and in the VC to validate methylation in the candidate genes identified in the 450k array (Illumina). In bold, CpGs from 450k array. Normal type, consecutive CpGs.
