# Supplementary material for: A two-gene epigenetic signature for the prediction of response to neoadjuvant chemotherapy in triple-negative breast cancer patients
Source: Clin Epigenetics. 2019 Feb 20;11:33. doi: 10.1186/s13148-019-0626-0 (PMC6381754; doi:10.1186/s13148-019-0626-0)
Supplement: Supplementary file 5 — Representation of the pathway interaction network of FERD3L and TRIP10 with other genes using Pathway Commons. FERD3L and TRIP10 are able to interact with different genes that have shown to be implicated in cancer drug resistance (PPT 452 kb) [file 13148_2019_626_MOESM5_ESM.ppt]

## Slide 1
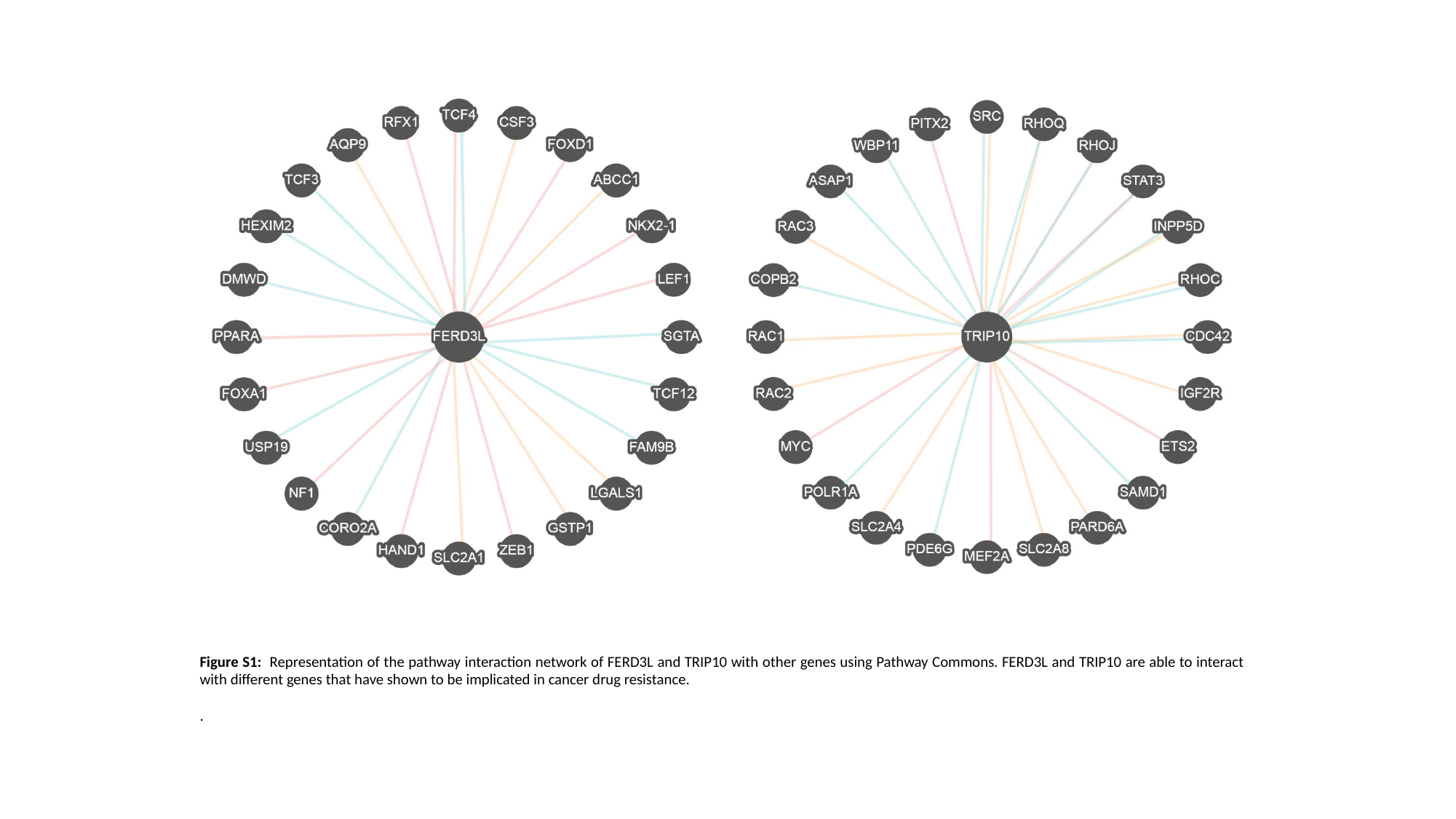

Figure S1: Representation of the pathway interaction network of FERD3L and TRIP10 with other genes using Pathway Commons. FERD3L and TRIP10 are able to interact with different genes that have shown to be implicated in cancer drug resistance.
.
