# Supplementary material for: A two-gene epigenetic signature for the prediction of response to neoadjuvant chemotherapy in triple-negative breast cancer patients
Source: Clin Epigenetics. 2019 Feb 20;11:33. doi: 10.1186/s13148-019-0626-0 (PMC6381754; doi:10.1186/s13148-019-0626-0)
Supplement: Supplementary file 6 — Mean differences in methylation levels according to clinicopathological prognostic factors in both cohorts (DC+VC). cT, clinical tumor size; cN, clinical nodule affectation (PPTX 48 kb) [file 13148_2019_626_MOESM6_ESM.pptx]

## Slide 1
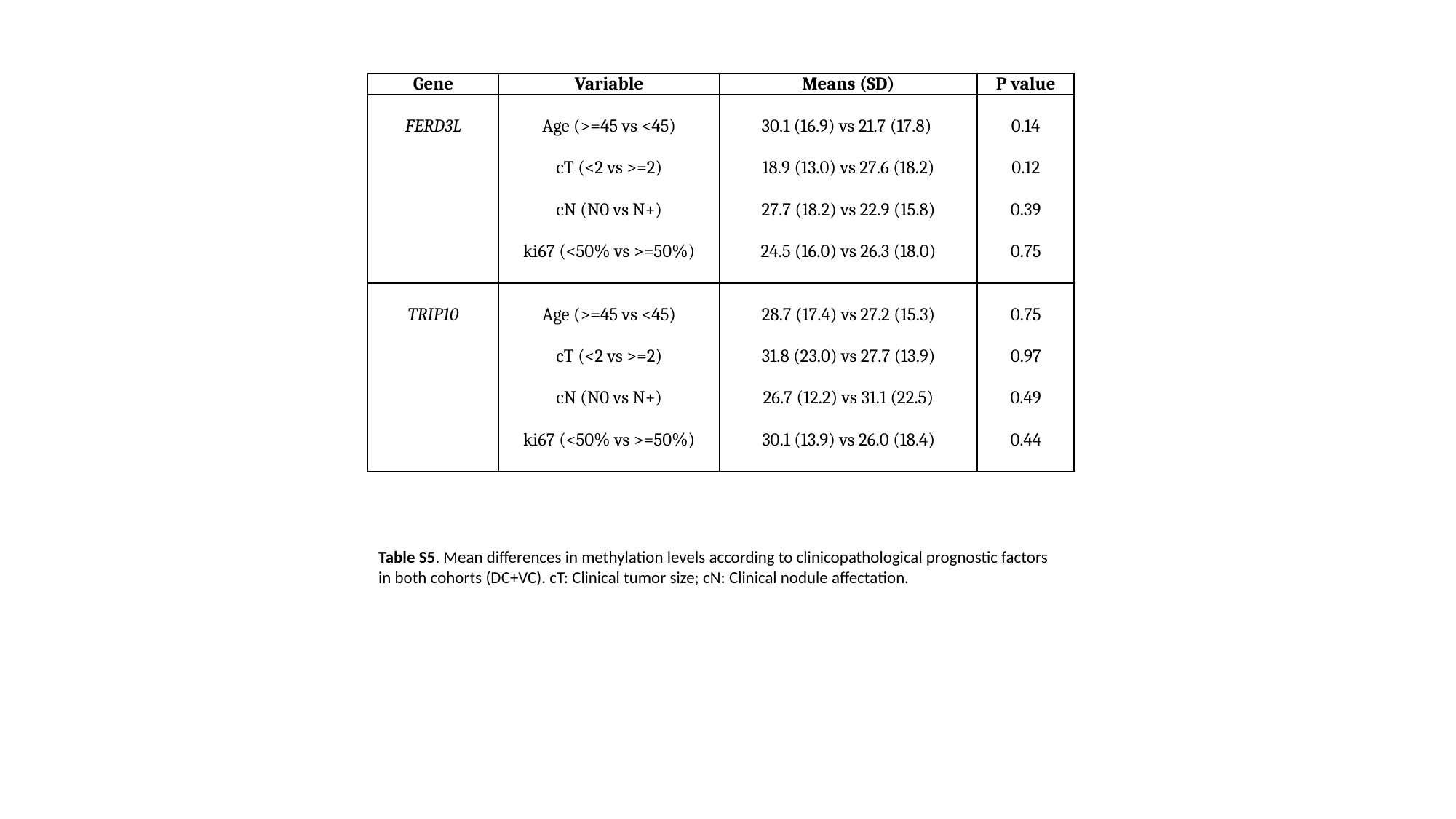

| Gene | Variable | Means (SD) | P value |
| --- | --- | --- | --- |
| FERD3L | Age (>=45 vs <45)   cT (<2 vs >=2)   cN (N0 vs N+)   ki67 (<50% vs >=50%) | 30.1 (16.9) vs 21.7 (17.8)   18.9 (13.0) vs 27.6 (18.2)   27.7 (18.2) vs 22.9 (15.8)   24.5 (16.0) vs 26.3 (18.0) | 0.14   0.12   0.39   0.75 |
| TRIP10 | Age (>=45 vs <45)   cT (<2 vs >=2)   cN (N0 vs N+)   ki67 (<50% vs >=50%) | 28.7 (17.4) vs 27.2 (15.3)   31.8 (23.0) vs 27.7 (13.9)   26.7 (12.2) vs 31.1 (22.5)   30.1 (13.9) vs 26.0 (18.4) | 0.75   0.97   0.49   0.44 |
Table S5. Mean differences in methylation levels according to clinicopathological prognostic factors in both cohorts (DC+VC). cT: Clinical tumor size; cN: Clinical nodule affectation.
