# Supplementary material for: A two-gene epigenetic signature for the prediction of response to neoadjuvant chemotherapy in triple-negative breast cancer patients
Source: Clin Epigenetics. 2019 Feb 20;11:33. doi: 10.1186/s13148-019-0626-0 (PMC6381754; doi:10.1186/s13148-019-0626-0)
Supplement: Supplementary file 7 — Clinical inclusion and exclusion criteria followed to select TNBC patients for the methylation study (PPT 89 kb) [file 13148_2019_626_MOESM7_ESM.ppt]

## Slide 1
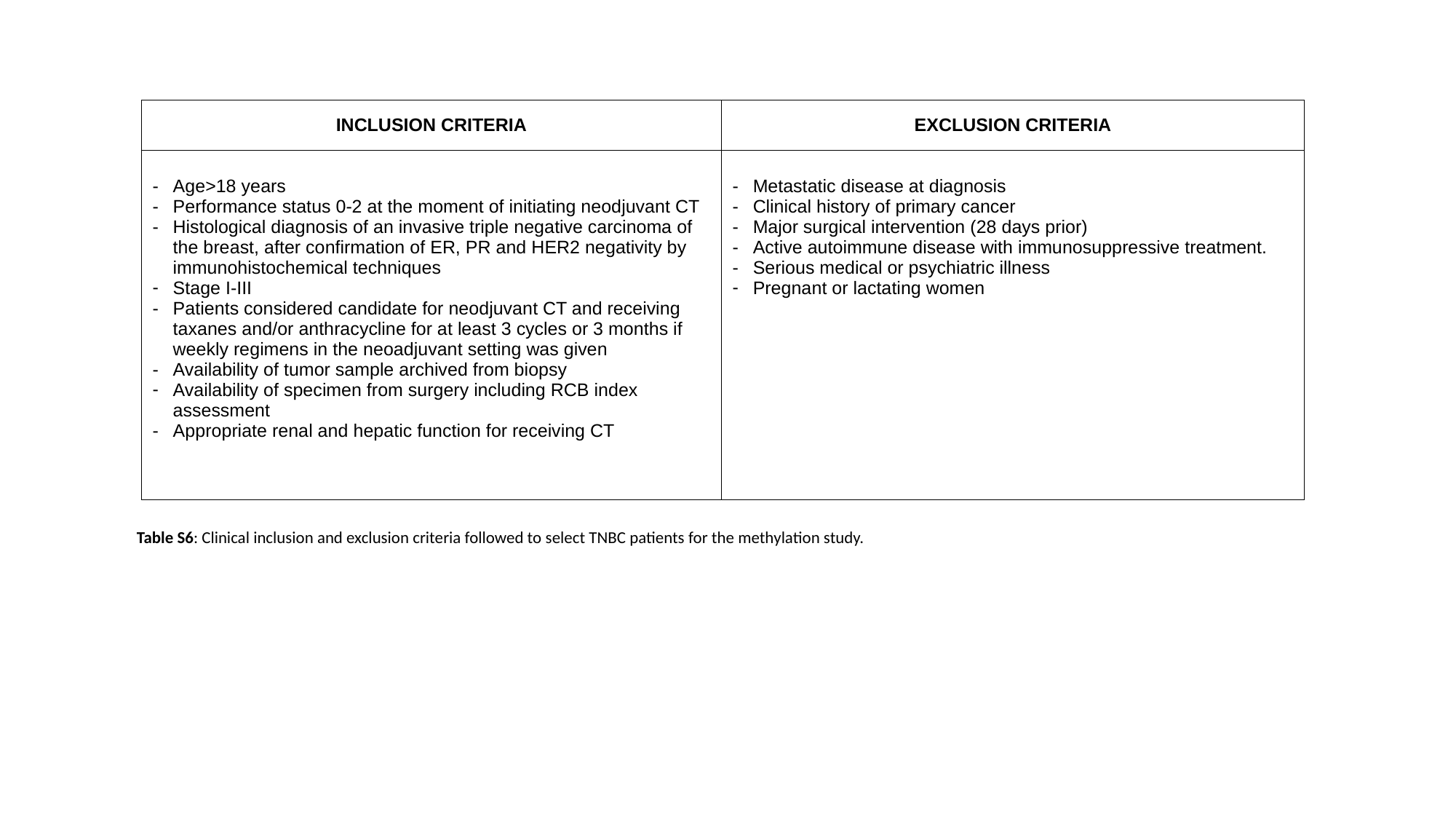

| INCLUSION CRITERIA | EXCLUSION CRITERIA |
| --- | --- |
| Age>18 years Performance status 0-2 at the moment of initiating neodjuvant CT Histological diagnosis of an invasive triple negative carcinoma of the breast, after confirmation of ER, PR and HER2 negativity by immunohistochemical techniques Stage I-III Patients considered candidate for neodjuvant CT and receiving taxanes and/or anthracycline for at least 3 cycles or 3 months if weekly regimens in the neoadjuvant setting was given Availability of tumor sample archived from biopsy Availability of specimen from surgery including RCB index assessment Appropriate renal and hepatic function for receiving CT | Metastatic disease at diagnosis Clinical history of primary cancer Major surgical intervention (28 days prior) Active autoimmune disease with immunosuppressive treatment. Serious medical or psychiatric illness Pregnant or lactating women |
Table S6: Clinical inclusion and exclusion criteria followed to select TNBC patients for the methylation study.
