# Supplementary material for: A two-gene epigenetic signature for the prediction of response to neoadjuvant chemotherapy in triple-negative breast cancer patients
Source: Clin Epigenetics. 2019 Feb 20;11:33. doi: 10.1186/s13148-019-0626-0 (PMC6381754; doi:10.1186/s13148-019-0626-0)
Supplement: Supplementary file 8 — Sequence of primers used by pyrosequencing in the validation assay of candidate genes obtained from 450k array (PPT 143 kb) [file 13148_2019_626_MOESM8_ESM.ppt]

## Slide 1
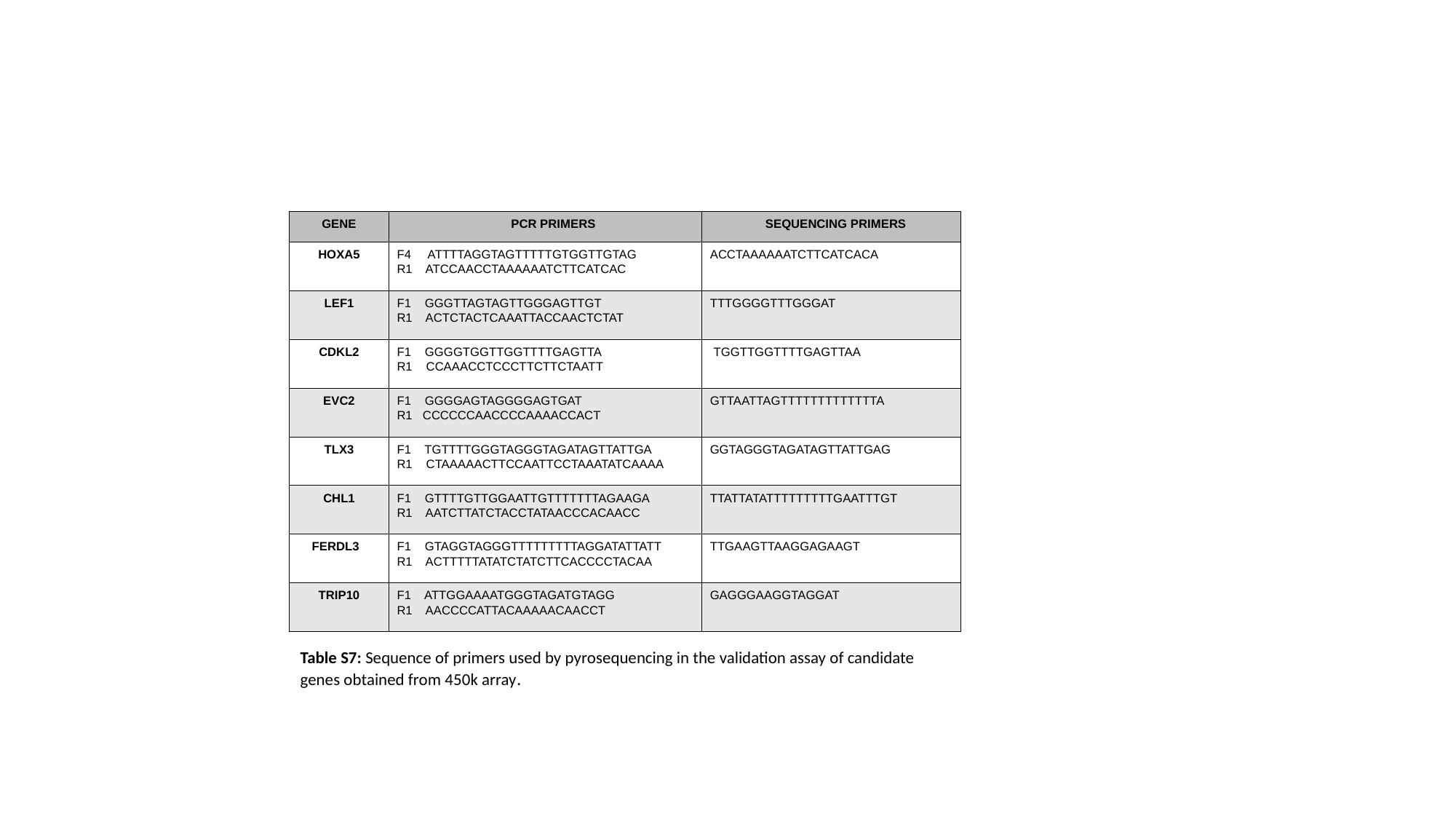

| GENE | PCR PRIMERS | SEQUENCING PRIMERS |
| --- | --- | --- |
| HOXA5 | F4 ATTTTAGGTAGTTTTTGTGGTTGTAG R1 ATCCAACCTAAAAAATCTTCATCAC | ACCTAAAAAATCTTCATCACA |
| LEF1 | F1 GGGTTAGTAGTTGGGAGTTGT R1 ACTCTACTCAAATTACCAACTCTAT | TTTGGGGTTTGGGAT |
| CDKL2 | F1 GGGGTGGTTGGTTTTGAGTTA R1 CCAAACCTCCCTTCTTCTAATT | TGGTTGGTTTTGAGTTAA |
| EVC2 | F1 GGGGAGTAGGGGAGTGAT R1 CCCCCCAACCCCAAAACCACT | GTTAATTAGTTTTTTTTTTTTTA |
| TLX3 | F1 TGTTTTGGGTAGGGTAGATAGTTATTGA R1 CTAAAAACTTCCAATTCCTAAATATCAAAA | GGTAGGGTAGATAGTTATTGAG |
| CHL1 | F1 GTTTTGTTGGAATTGTTTTTTTAGAAGA R1 AATCTTATCTACCTATAACCCACAACC | TTATTATATTTTTTTTTGAATTTGT |
| FERDL3 | F1 GTAGGTAGGGTTTTTTTTTAGGATATTATT R1 ACTTTTTATATCTATCTTCACCCCTACAA | TTGAAGTTAAGGAGAAGT |
| TRIP10 | F1 ATTGGAAAATGGGTAGATGTAGG R1 AACCCCATTACAAAAACAACCT | GAGGGAAGGTAGGAT |
Table S7: Sequence of primers used by pyrosequencing in the validation assay of candidate genes obtained from 450k array.
